# A Realist Scoping Review of Community Nutrition Interventions in the UK: Implications for the ‘Nutrition Skills for Life’ Programme

**DOI:** 10.1111/jhn.70008

**Published:** 2025-01-08

**Authors:** J. Lisa Williams, Carolyn Wallace, Teresa Filipponi

**Affiliations:** ^1^ Department of Community Nutrition and Dietetics Cardiff and Vale University Health Board Cardiff UK; ^2^ Faculty of Life Sciences and Education University of South Wales Pontypridd UK; ^3^ PRIME Centre Wales Cardiff UK; ^4^ Wales School for Social Prescribing Research (WSSPR) Pontypridd UK

**Keywords:** community intervention, dietetics, inequities, nutrition, prevention, programme theory, realist, socioeconomic disadvantage

## Abstract

**Background:**

Nutrition Skills for Life (NSFL) provides training and support for communities and organisations to implement Community Nutrition Interventions (CNIs) that meet identified needs. To inform future NSFL evaluation, this scoping review, using a realist approach sought to determine the underpinning initial programme theory (IPT) for how CNIs support socioeconomically disadvantaged (SED) communities to access a healthy diet, as detailed in the protocol doi.org/10.17605/OSF.IO/D56FK.OSF.IO/D56FK.

**Methodology:**

Reporting standards for realist syntheses (RAMESES) and scoping reviews (PRISMA‐ScR) were used. Four electronic databases and grey literature were searched. Of the 1920 documents identified, 45 were included in the analysis. Data relating to Context, Mechanism and Outcomes were extracted and presented as C‐M‐O configurations (CMOCs). Documents were assessed for relevance to the research question and usefulness in terms of their contribution towards the IPT.

**Results:**

The IPT, underpinned by the Ottawa Charter for Health Promotion, comprises 17 consolidated CMOCs. These are narratively discussed as follows: understanding community needs; consistent nutrition messages; knowledgeable, skilled, confident practitioners/facilitators and practising new skills.

**Conclusions:**

Realist research and analysis of CMOCs provided a deeper understanding of how CNIs can be implemented to support SED communities in accessing a healthy diet. Interventions ‘worked’ when they acknowledged and addressed identified barriers to healthy eating, provided reliable, trusted, easy‐to‐understand nutrition messages, were delivered by confident, knowledgeable practitioners, and facilitated strategies such as meal preparation. Further realist evaluation to refine the IPT could inform the evaluation of other complex public health interventions.

## Summary

- The complex factors that interconnect to influence people's diets bring challenges to evaluating Community Nutrition Interventions (CNIs).
- A realist approach to evaluating complex interventions, seeks to go beyond questions about whether a programme works, to ask what works for whom, in what contexts, in what respects and how and to develop programme theory.
- To our knowledge, a programme theory for what works, for whom and under what circumstances for CNIs in SED communities has not been determined in the UK.
- A complex, multi‐layered combination of contexts and mechanisms at the individual (intra and interpersonal factors), social and cultural, and physical environment levels impact dietary behaviours.

## 1 Introduction

Globally, poor diet and obesity are the leading risk factors for mortality and morbidity related to non‐communicable diseases (NCDs) [1]. Dietary risk factors include low intakes of whole grains, fruit, vegetables, nuts, seeds and oily fish, and high intakes of saturated fat, free sugars and salt [1, 2]. In Wales, 61% of adults are living with either overweight or obesity [3]. These dietary risk factors and the prevalence of obesity are greater among those from more disadvantaged backgrounds [4, 5]. It is estimated that overweight and obesity costs the Welsh National Health Service (NHS) £86 million per year [6]. The impact of dietary factors extends beyond the burden of disease, with direct implications for planetary health and resource sustainability [7].

There is clear evidence of a social gradient whereby people living in areas with more disadvantage have poorer health [8, 9]. Inequalities in diets contribute to overall inequalities in health, defined as systematic differences in health between groups, which are judged as unfair and avoidable [10]. No single intervention can halt the rising levels of obesity and health inequalities on its own [11, 12]. However, adopting the five action areas of the Ottawa Charter's framework for health promotion, such as creating supportive environments and developing personal skills, when planning interventions, can increase effectiveness [13, 14]. Indeed, comprehensive strategies involving multiple interventions at multiple levels that influence human behaviour, increasingly referred to as whole system approaches, are needed to address this complex public health challenge [15, 16].

**Figure 1:**
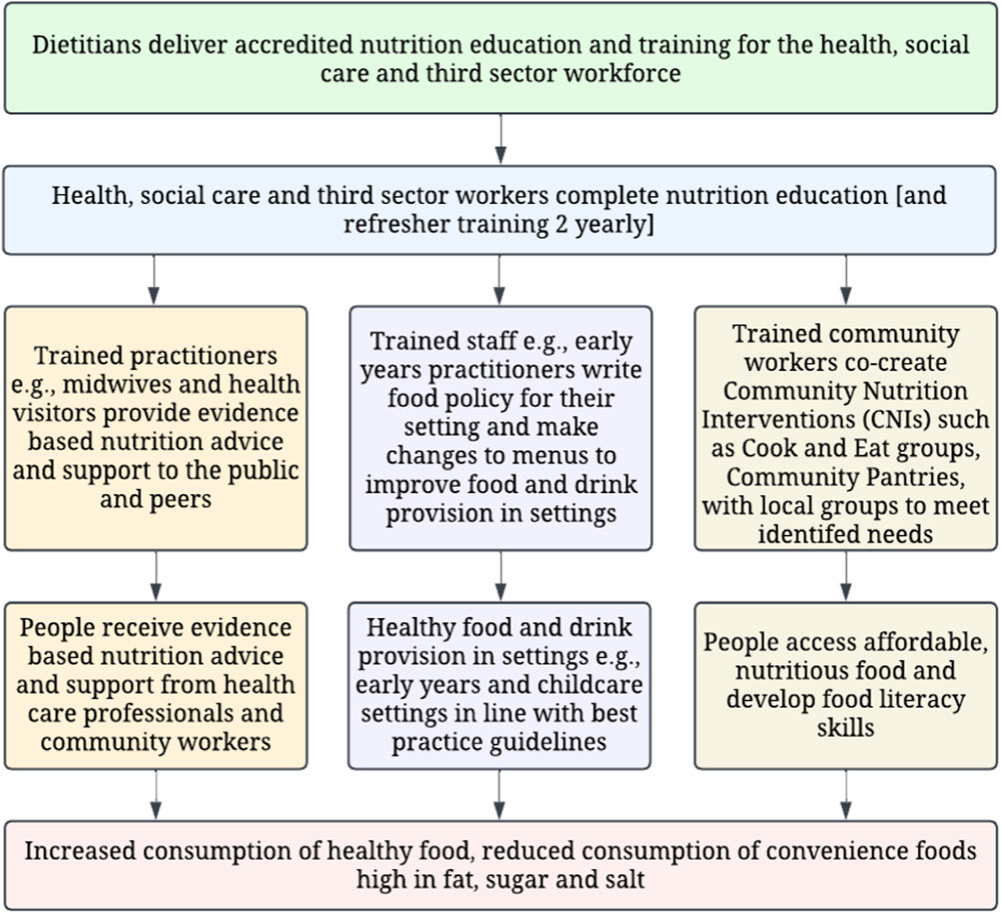
Nutrition Skills for Life: Nutrition training and capacity development.

The Nutrition Skills for Life (NSFL) programme in Wales, co‐ordinated by NHS dietitians, provides nutrition education and training for community‐based practitioners so that they can embed evidence‐based nutrition practices into their work. The programme focuses on reducing diet‐related inequalities by empowering people, communities and organisations to overcome barriers to accessing affordable, nutritious and sustainable food. Underpinned by the Ottawa Charter framework, the programme aims to support the development of effective and sustainable community nutrition interventions (CNIs) that enable people and communities to eat a healthy and sustainable diet [17]. CNIs or healthy eating strategies have been defined as ‘an organised effort intended to result in significant and sustainable changes in the dietary behaviours of an identified group and/or entire population' [18]. The nutrition education and capacity‐building model implemented by NSFL is summarised in Figure 1. Examples of CNIs that NSFL supports include healthcare professionals' (HCPs) brief interventions, healthy food provision in community settings and the development of community cooking groups.

Promising strategies for improving the diets of low‐income groups include addressing structural barriers that can impact diet quality, such as affordability, convenience and accessibility of healthy foods, and empowering individuals and communities to make informed food choices, for example, through nutrition education and improved food literacy [12, 18, 19, 20]. Food literacy is a collection of interrelated knowledge, skills and behaviours required to plan, manage, select, prepare and eat foods to meet needs. It is the scaffolding that empowers individuals, households, communities or nations to protect diet quality through change and support dietary resilience over time [21]. Food literacy may be a means to enable people to navigate the current food system and make healthy food decisions to meet food and nutrition needs [21, 22].

However, the complex individual, socio‐cultural and environmental factors that interconnect to influence the diets of low‐income communities bring challenges to evaluating CNIs [23]. A new framework for developing and evaluating complex interventions emphasises the need for greater attention to understanding how and under what circumstances interventions bring about change [24]. The framework draws attention to the value of identifying mechanisms of change. Mechanisms are the causal links between contextual factors and intervention components that determine and shape whether and how outcomes are generated [23, 24]. Applying this to CNIs, evaluation should determine what contextual factors are needed for CNIs to generate mechanisms that can bring about change in dietary behaviour. This review therefore draws upon a realist logic of enquiry, a theory‐driven approach to the evaluation of complex interventions that seeks to go beyond questions about whether a programme works, to ask ‘what works for whom, in what contexts, in what respects and how.’ A realist approach aims to theorise what it is about a programme that might cause change and to develop programme theory [25]. The programme theory is commonly defined as the set of assumptions of programme designers (or other actors involved) that explain how and why they expect the intervention to reach its objective(s) and in which conditions [26]. If certain resources (material, social, cognitive) are provided (the intervention) in a particular context (C), then they will ‘trigger’ participants’ reasoning (the mechanism (M)), generating a change or improvement that impacts their nutrition (outcome (O)) [27]. The CMO configuration (CMOC) is the heuristic suggested by Pawson and Tilley and used by some realists during analysis to identify the causal links between context, mechanism and outcome [26]. CMOCs are developed and used to build plausible arguments for how programmes might work. A review of realist research used to evaluate public health nutrition interventions concluded that this approach is well‐placed to deal with the complexities of dietetic practice [28]. The approach generates rich explanatory findings that can provide important insights, which add to our knowledge about how and for whom CNIs, such as those facilitated by NSFL in Wales, ‘work’ and how they can be adapted to best meet local needs [29].

The aim of this scoping review is, therefore, to identify and map the evidence and answer the question, how do CNIs support people, particularly those from socioeconomically disadvantaged (SED) communities, to access a healthy diet? The objectives of the review are:

- To begin to determine the underpinning initial programme theory (IPT) for how CNIs work (or don't work).
- To identify specific programme theory that may warrant a full realist review, or realist evaluation, to test and refine the theory and inform the future direction of the NSFL programme in Wales.

To our knowledge, a programme theory for what works, for whom and under what circumstances for CNIs in SED communities, has not been determined in the UK. In undertaking this review, we aim to provide insights that are useful for others working in public health nutrition and dietetics.

## 2 Methods

A scoping review was deemed appropriate as they are used to address broad research questions and to map evidence from a variety of sources [30]. The results can provide indications of research gaps and inform new research projects [31]. A realist approach was utilised to analyse the data sources, identify programme theories (CMOCs) [32] and meet the review's objectives. Other reviews have used a combination of scoping review and realist approach, where the research question and synthesis were strongly informed by realist philosophy and review methods [33, 34].

### 2.1 Protocol and Registration

This review is reported in accordance with the Realist And Meta‐narrative Evidence Syntheses: Evolving Standards (RAMESES) for realist synthesis [32] and the Preferred Reporting Items for Systematic Reviews and Meta‐Analyses extension for Scoping Reviews (PRISMA‐ScR) checklist [30] (see Supporting Information: File 1). The review was conducted between July 2022 and September 2023 and the protocol was registered on the Open Science Framework [35]. Changes from the protocol included a second reviewer screened titles and abstracts for a random sample of 10% of citations; studies from systematic reviews that met the inclusion criteria were included, and the data extraction template was modified to include reference to substantive theory and details of specific contexts, mechanisms and outcomes.

### 2.2 Eligibility/Inclusion Criteria

Included documents were published in English, conducted in the UK between January 2012 and August 2022, focused on adults (19+ years) and referred to concepts, such as diet and nutrition, community interventions, practical cooking skills and food literacy, dietary behaviour and food access and security (see Supporting Information: File 2 for PCC table and eligibility criteria). Documents were excluded if the CNIs were school‐based and did not include parent or carer involvement; were designed to treat existing conditions, for example, diabetes, dysphagia, protein‐energy malnutrition or focussed on single nutrients rather than whole diet approaches.

### 2.3 Search Strategy, Screening and Data Extraction

CINAHL, Medline (Ovid), PsycINFO and Social Care Online (SCIE) databases were searched using keywords and search terms (see Supporting Information: File 2), which were determined with expert help from librarians. Google Scholar was searched for relevant grey literature. All identified citations were uploaded into the Zotero reference management system and duplicates were removed. Screening by title was undertaken against the pre‐specified inclusion and exclusion criteria. Relevant sources were retrieved in full, and their citation details were imported into Rayyan citation management software for abstract screening.

Data were extracted from the included documents and recorded in Microsoft Excel according to pre‐determined document characteristics (see Supporting Information: File 2). CMOCs were formulated as ‘If–then’ statements to clarify the relationship and potential causal mechanism that was triggered under specific contexts to bring about outcomes. CMOCs were coded and grouped according to patterns and themes to build coherent arguments, which could contribute towards developing the IPT [26]. CMOCs were integrated to produce the most economical expressions of similar CMOCs as consolidated CMOCs. The process described by Pearson et al. [36] was followed to integrate and consolidate CMOCs according to similar outcomes or mechanisms. For CMOCs that were not new but added important refinements to the consolidated CMOC, the refinement was added verbatim. The final consolidated CMOCs were expressed as “if…then…because” statements to clearly articulate the configuration, the mechanism activated and the context in which the mechanism was contingent [37].

## 3 Results

### 3.1 Selection of Sources of Evidence

**Figure 2:**
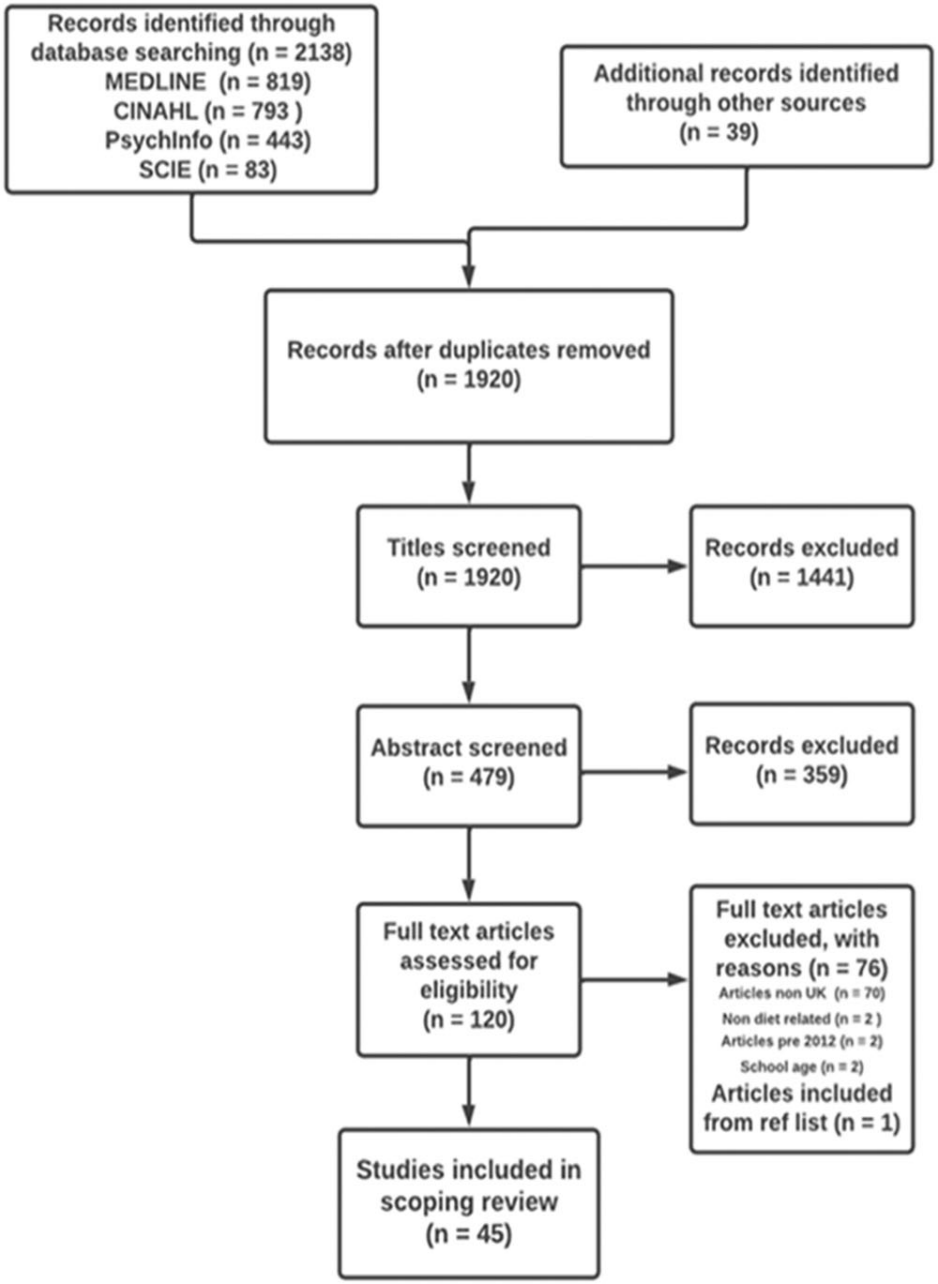
Preferred Reporting Items for Systematic Reviews and Meta‐Analyses (PRISMA) diagram of document selection.

In total, 2138 documents were identified through database and grey literature searching. Following de‐duplication, 1920 documents were screened by title and abstract, and 120 were selected for full read. Forty‐five documents were selected for the review [38, 39, 40, 41, 42, 43, 44, 45, 46, 47, 48, 49, 50, 51, 52, 53, 54, 55, 56, 57, 58, 59, 60, 61, 62, 63, 64, 65, 66, 67, 68, 69, 70, 71, 72, 73, 74, 75, 76, 77, 78, 79, 80, 81, 82] (see Figure 2 for the PRISMA diagram for document selection). Documents were assessed for relevance to the research question and usefulness in terms of their contribution towards determining the IPT. Documents were rated ‘low’ (*n* = 3) if they did not contribute any relevant CMOCs, ‘moderate’ (*n* = 7) if they contributed to 1 CMOC and ‘high’ (*n* = 35) if they contributed to 2 or more CMOCs (see Supporting Information: Files 3 and 4).

### 3.2 Characteristics of Sources of Evidence

Most of the included documents were qualitative studies (*n* = 23; 51.1%), followed by quantitative studies (*n* = 11; 24.4%) and mixed methods (*n* = 9; 20%) or reviews (*n* = 2; 4%) (see Supporting Information: File 4). Most documents used multiple research methods (*n* = 24; 53.3%), followed by interview only (*n* = 11; 24.4%), survey/questionnaire (*n* = 6; 13.3%) and focus group (*n* = 2; 4.4%) (see Tables 1 and 2). Most studies were conducted in England (*n* = 31; 69%) followed by Northern Ireland/Ireland (*n* = 4; 9%), Wales (*n* = 2; 4%) and Scotland (*n* = 1; 2%). Eleven documents (24%) reported evaluation findings from specific community health programmes or CNIs [45, 50, 55, 56, 57, 62, 64, 72, 75, 78, 81]. Eleven documents focussed on the impact of socioeconomic status on food intake [41, 42, 45, 48, 60, 62, 68, 69, 72, 76, 79].

**Table 1:** Characteristics of sources of evidence.

**Study/article characteristics**	***n*/45 (%)**	**Study/article characteristics**	***n*/45 (%)**	**Study/article characteristics**	***n*/45 (%)**
**Type of evidence source**		**Part of UK study was conducted**		**Intervention place/setting**	
Research article	43 (95.6)	England	31 (68.9)	Not setting based	19 (42.2)
‘In practice’ article	1 (2.2)	Northern Ireland or Island of Ireland	4 (8.9)	Routine healthcare, e.g., antenatal	8 (17.8)
Evaluation reports	1 (2.2)	Wales	2 (4.4)	Early years‐children's/family centres	2 (4.4)
		Scotland	1 (2.2)	Schools	2 (4.4)
				Youth	1 (2.2)
		Multiple UK sites	4 (8.9)	Community	7 (15.6)
**Evaluation of a specified community nutrition intervention**		UK combined with a non‐UK site	2 (4.4)	Workplace	1 (2.2)
No	34 (75.6)	N/A systematic review	1 (2.2)	Food retail outlets	1 (2.2)
Yes	11 (24.4)			Older adult's care	4 (8.9)
**Research design approach**		**Behaviour change techniques/models**		**Life course stage/population subgroup**	
Quantitative	11 (24.4)	Referred to 1 technique/model	8 (17.8)	Pregnancy/post‐natal	7 (15.6)
Mixed method	9 (20)	Referred to 2 techniques/models	3 (6.7)	Parents of infants and young children	8 (17.8)
Reviews	2 (4.4)	**Techniques/models**			
		‐ Motivational interviewing	3 (6.7)	Parents of school‐aged children	3 (6.7)
**Specific focus on SED**		‐ Goal setting	7 (15.6)	Young People/care experienced	2 (4.4)
Yes	11 (24.4)	‐ COM‐B model	2 (4.4)	Adults, including vulnerable groups	19 (42.2)
No	34 (75.6)	‐ Logic model/Theory of change	2 (4.4)	Older adults	6 (13.3)
**Methods of data collection**		**Theoretical basis for intervention**		**Staff groups that deliver CNIs**	
Multiple methods	24 (53.3)	Not identified	33 (73.3)		
Interviews	11 (24.4)	Referred to 1 theory	9 (20)	Not specified	21 (46.7)
Survey/questionnaire	6 (13.3)	Referred to 2 theories	3 (6.7)	Midwives/Health Visitors	7 (15.6)
Focus groups	2 (4.4)	**Theories**		School‐based staff	1 (2.2)
N/A	2 (4.4)	‐ Social cognitive theory	3 (6.7)	Childcare centre staff	2 (4.4)
**Level of intervention**		‐ Self‐efficacy theory	2 (4.4)	Youth workers	1 (2.2)
Individual (intra/interpersonal)	13 (28.9)	‐ Ecological systems theory	2 (4.4)	Nutritionist/community food worker	2 (4.4)
Socio/cultural environment	20 (44.4)	‐ Self‐determination theory	2 (4.4)	Trained chefs/community tutors	4 (8.9)
Physical environment	12 (26.7)	‐ Practice‐theoretical/practice‐oriented approach	2 (4.4)	Nurses, carers, housing association staff	4 (8.9)
		‐ Theoretical domains framework	1 (2.2)	Environmental health officers	1 (2.2)
		‐ Theory of planned behaviour	2 (4.4)	Food retailers	1 (2.2)
		‐ Attachment theory	1 (2.2)	Voluntary organisations	1 (2.2)

**Table 2:** Methodology, research design and methods.

**Methodology (*n*** = **45)**	**Study design (*n*** = **45)**	**Research methods (*n*** = **45)**
Qualitative (*n* = 23)	Exploratory (*n* = 17) [38, 40, 41, 42, 43, 47, 51, 52, 59, 60, 63, 67, 68, 70, 71, 76, 82]	Interviews (*n* = 8)
		Focus groups (*n* = 2)
		Interviews + focus groups (*n* = 4)
		Interviews + Photovoice (*n* = 1)
		Interviews + observation (*n* = 1)
		Focus groups + interviews + observations + documentary analysis (*n* = 1)
	Evaluation (*n* = 6) [45, 50, 62, 72, 78, 81]	Interviews (*n* = 3)
		Interviews + focus group (*n* = 1)
		Interviews, focus group + documentary analysis (*n* = 1)
		Focus groups + workshops + questionnaire (*n* = 1)
Quantitative (*n* = 11)	Cross sectional (*n* = 7) [39, 46, 49, 54, 58, 77, 79]	Questionnaire(s) (*n* = 3)
		Questionnaires + FFQ/24 h recall (*n* = 1)
		Survey (*n* = 2)
		Survey + diet recall/food diary (*n* = 1)
	Randomised Control (*n* = 2) [67, 80]	Questionnaire (*n* = 1)
		Questionnaire + food diary (*n* = 1)
	Cohort (*n* = 2) [61, 73]	Survey + food frequency questionnaire + clinical measurements (*n* = 2)
Mixed method (*n* = 9)	Exploratory (*n* = 3) [65, 69, 74]	Survey, focus group + interviews (*n* = 1)
	Evaluation (*n* = 5) [55, 56, 57, 64, 75]	Survey, FFQ + focus group (*n* = 1)
	Comparative case study (*n* = 1) [48]	Survey with qual. & quan. component (*n* = 1)
		Pre/post survey + follow‐up focus groups/qual. questions (*n* = 2)
		Survey/questionnaire + interviews/qual. questions (*n* = 2)
		Interviews + observations + survey + documentary analysis (*n* = 1)
		Interviews + observations + survey (*n* = 1)
Review (*n* = 2)	Systematic review (*n* = 1) [66]	N/A (*n* = 2)
	Narrative piece (*n* = 1) [53]	

The types of CNIs included nutrition brief interventions during pregnancy/postnatally [39, 50, 58], practical cooking skills interventions [44, 46, 55, 57, 61, 67] and healthy food provision in settings [49, 56, 64, 70, 72, 81]. Factors influencing the food decisions of SED communities were explored in three documents [41, 42, 60]. Twelve documents referred to substantive theories [40, 47, 48, 50, 55, 66, 67, 68, 75, 77, 79, 81]. Bandura's social cognitive theory [83] was the most commonly cited [55, 68, 79]. Goal setting was the most frequently cited behaviour change technique [40, 50, 52, 66, 67, 76, 78].

### 3.3 Main Findings

**Figure 3:**
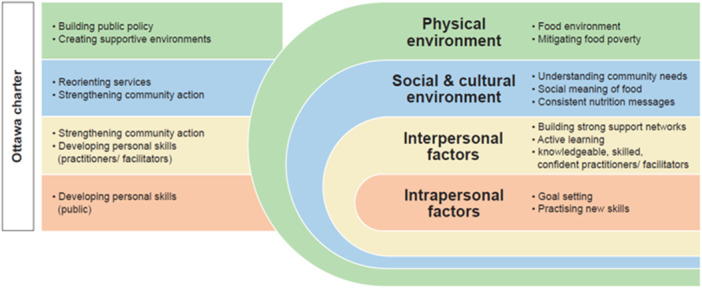
Ten broad classifications of extracted C‐M‐O configurations (CMOCs).

The CMOCs (*n* = 170) extracted from the data sources were grouped as 10 broad areas of interest, or classifications, for how CNIs might work particularly for SED communities (Figure 3). Based on socio‐ecological models, which illustrate the layers of influence on human behaviour [13, 16, 84, 85], these classifications represent a complex, multi‐layered combination of contexts and mechanisms at the individual (intra and interpersonal factors), social and cultural, and physical environment levels that impact dietary behaviours. These broad classifications align with the Ottawa Charter's five action areas, which conceptualises these layers of influence into a clear and widely applicable framework for public health programme planning and design [13]. This broader conceptual model is fully described elsewhere [86].

The classifications that were highly relevant to the NSFL programme and had the most associated CMOCs, were further refined and synthesised into consolidated CMOCs (*n* = 17). “If…then…because” phraseology was used to clearly articulate the configurations (Tables 3, 4, 5, 6). Each consolidated CMOC narrative is discussed below under the following headings: understanding community needs, consistent nutrition messages, knowledgeable, skilled, confident practitioners/facilitators and practising new skills. Data contributing to each consolidated CMOC can be found in Supporting Information: File 5. The programme theory will evolve into a final programme theory after further testing with programme stakeholders.

**Table 3:** Consolidated C‐M‐O configurations (CMOCs) 1–4 for understanding community needs.

Understanding community needs			
	IF (Context)	THEN (Outcome)	BECAUSE (Mechanism)
CMOC 1	**If** all organisations supporting vulnerable groups to improve eating patterns and nutritional status fully understand the factors that impact people's eating patterns and work collaboratively	**Then** nutritional interventions are more likely to meet individual/community needs and circumstances resulting in improved nutritional health and wellbeing	**Because** existing levels of knowledge, beliefs and priorities that influence decision‐making and food choices are acknowledged and more tailored, practical, trusted advice and support is provided.
CMOC 2	**If** interventions provide information about healthy food without engaging with the unequal contexts of everyday life that shape healthy food norms including structural issues	**Then** interventions are likely to have a limited impact	**Because** of a poor understanding of factors impacting people's eating patterns and missed opportunities to counteract identified issues.
CMOC 3	**If** staff planning and delivering community nutrition interventions with vulnerable community groups encourage discussion and interaction with community members	**Then** this can maximise engagement, ensure provision is culturally appropriate, increase people's self‐value, cooking self‐efficacy, general confidence, resilience and ability to mitigate some of the debilitating aspects of food poverty	**Because** local partnerships are built and relationships of trust are established, which instil trust and confidence in others to share advice and life experiences
CMOC 4	**If** HCPs who lack the belief that they were able to make a difference in people's diets receive training to improve their communication and behaviour change skills, knowledge and understanding of complex social and cultural determinants impacting upon individual circumstances and address their expressed prejudices related to stereotypes	**Then** this can support them to more effectively and inclusively meet people's health needs and facilitate dietary behaviour change	**Because** they critically reflect on their own emotions, attitudes and beliefs in relation to weight and shape, and gain knowledge, behaviour change skills and confidence.

**Table 4:** Consolidated C‐M‐O configurations (CMOCs) 5–8 for consistent nutrition messages.

Consistent nutrition messages			
	IF (Context)	THEN (Outcome)	BECAUSE (Mechanism)
CMOC 5	**If** people from vulnerable groups have incomplete knowledge of nutrition and do not receive reliable information and advice from trusted sources, despite a high desire to provide a healthy diet for themselves or their family	**Then** this can lead to poor adherence to dietary recommendations and a greater risk of poor nutritional intake,	**Because** of confusion, mistaken beliefs and commonly held perceptions about how and why to make healthier choices and seeking information from informal sources
CMOC 6	**If** healthcare professionals (HCPs) caring for vulnerable people have knowledge and understanding of people's nutritional needs and their level of existing knowledge and/or mistaken or false beliefs	**Then** this can ensure people receive correct information, do not receive conflicting messages, are not advised to follow unsafe practices and knowledge‐attitude gaps are addressed	**Because** HCPs can adapt dietary advice to meet individual's needs, correct nutrition information is available, and confusion from conflicting messages is avoided
	Conversely, i**f** HCPs have insufficient knowledge and understanding of people's nutritional needs	**Then** this can lead to unsafe practice and missed opportunities to facilitate behaviour change and identify and address knowledge‐attitude gap	**Because** correct nutrition information is unavailable and people feel unsupported
CMOC 7	**If** unhealthy foods were provided alongside health options in retail and public sector settings and were more affordable	**Then** conflicting messages had the potential to undermine efforts to encourage healthier food consumption behaviour	**Because** of confusion due to the food provided not aligning with nutritional guidelines, unhealthy options being considered ‘a treat’ and the perception that healthy foods are less affordable.
	Conversely, **if** healthy foods were cooked alongside staff during on‐site cooking sessions to cook full meals and healthy foods were more affordable	**Then** this can minimise conflicting messages that undermine efforts to encourage healthier food consumption behaviour	**Because** the food provided aligns with nutritional guidelines, nutrition education sessions are embedded and the perception that healthy foods are unaffordable is reduced.
CMOC 8	**If** nutrition education and practical cooking skills development opportunities are provided at life stages when people are most receptive (e.g., early parenthood, young adults, people with long‐term conditions) including digital technology and social media interventions/resources	**Then** this can facilitate dietary behaviour change, provide ‘the tools needed for a healthy lifelong relationship with food’, encourage the adoption of healthy cooking habits into daily routines and manage food waste well, which can influence food spending	**Because of** increased motivation to feed their family a nutritious diet, improvements in food literacy ‐ learning about nutrition and the importance of a balanced diet for health, learning practical food preparation skills and new recipes, understanding food labels, portion sizes and ways to encourage children to eat well

**Table 5:** Consolidated C‐M‐O configurations (CMOCs) 9–12 for knowledgeable, skilled, confident practitioners/facilitators.

Knowledgeable, skilled, confident practitioners/facilitators			
	IF (Context)	THEN (Outcome)	BECAUSE (Mechanism)
CMOC 9	**If** healthcare practitioners receive training and education in nutrition, behaviour change techniques, such as motivational interviewing, having healthy conversations and goal setting	**Then** they are more likely to feel better equipped to discuss eating habits, provide dietary advice and support behaviour change for vulnerable community groups	**Because** they are more confident in their nutrition knowledge, have greater self‐confidence and self‐efficacy to raise sensitive topics, are empowered with skills to take a structured approach to conversations and are motivated to discuss health behaviours.
CMOC 10	**If** healthcare practitioners caring for people living with obesity are motivated but lack the confidence to raise the topic of weight	**Then** they might avoid discussing eating habits	**Because** they are anxious and fearful of coming across as judgemental and lacking specialist knowledge of how to address eating, offending and causing upset, and negatively impacting their relationship with women.
	Conversely, **if** training is provided to address knowledge gaps for healthcare practitioners who care for people living with obesity	**Then** this can increase the number of healthy conversations and improve care and support provided for vulnerable groups	**Because** this can give staff greater confidence and self‐efficacy to raise the topic, alleviate anxiety about discussing weight, reduce fear of coming across as judgemental and lacking specialist knowledge of how to address eating and could positively impact their relationship with women.
CMOC 11	**If** families are supported with positive, practical messages about healthy eating, delivered by confident knowledgeable and skilled professionals who encourage people to set their own SMART goals for dietary change that they are able to achieve	**Then** this supports behaviour change for dietary improvement in the early years	**Because** the advice is regarded as reliable and trustworthy, raises people's confidence and self‐efficacy to make incremental dietary changes, and gives a sense of ownership over the goals and accomplishment and pride when goals are met.
	Conversely, **if** families do not receive this support	**Then** behaviour change is not supported	**Because** of their incomplete knowledge or mistaken beliefs from informal sources and insufficient knowledge and support from HCPs
CMOC 12	**If** staff caring for older adults receive training to provide knowledge and understanding of the nutritional needs of older people who are at risk of undernutrition, are alert for signs of malnutrition and are able to offer older adults and their relatives advice and support	**Then** this can contribute towards the provision of good nutritional care	**Because** commonly held perceptions are addressed, confusion over conflicting messages is avoided, dietary advice can be appropriately adapted to meet individuals' needs and relatives can proactively contribute.

**Table 6:** Consolidated C‐M‐O configurations (CMOCs) 13–17 for practising new skills.

Practising new skills			
	IF (Context)	THEN (Outcome)	BECAUSE (Mechanism)
CMOC 13	**If** people had opportunities to socialise with others, practise and experience meal planning, preparation, cooking, storing and tasting new foods/dishes	**Then** this can result in wasting less food, saving time and money preparing and cooking food, increased cooking from raw ingredients and reduced intake of HFSS convenience/fast foods, healthier food choices and better‐quality diet	**Because** of increased knowledge, skills, confidence, enjoyment and self‐efficacy in the ability to cook home‐prepared meals.
CMOC 14	**If** practical cooking activities for families include parents (e.g., mothers as the ‘gatekeeper’ of food provision in the home), teach the importance of planning ahead, using leftover ingredients and batch cooking and focus on developing children and young people's food and cooking skills	**Then** participants are more likely to feel confident to experiment, prepare a wider repertoire of dishes from scratch, avoid food waste, improve healthy eating at the family level and support better diet quality outcomes for children that track through to adult life	**Because** of greater self‐efficacy, knowledge, skills and confidence to cook home‐prepared meals, ability to shop more thriftily and easily adjust recipes to meet family preferences and possibly increased food and health motivation and cooking identity of children and young people.
CMOC 15	**If** people are provided with information alongside the opportunity to practise cooking and participate in fun activities	**Then** this reduces reliance on HFSS convenience foods and promotes increased consumption of healthier, unprocessed foods	**Because** of enjoyment and pleasure from cooking, reduced stress when involving young children, reduced risk that family members reject food and increased intention to cook from basic ingredients.
CMOC 16	**If** people have an opportunity to experiment and cook new dishes and have a repertoire of well‐known dishes	**Then** this increases the likelihood of cooking from scratch at home and consuming healthier, unprocessed foods	**Because** it helps to eliminate the fear of wasting food and money instilled by previous personal disasters in the kitchen, improves confidence and reduces the risk of rejection when new dishes are served
	Conversely, **if** families on a low income do not have a safe space for experimenting and learning [eroded due to financial constraints] with food preparation and cooking	**Then** they are less likely to repeatedly practice developing new skills to prepare minimally processed foods	**Because** they fear failure or the risk of rejection when serving food, leading to wasting food and money
CMOC 17	**If** people from vulnerable groups lack knowledge of what constitutes healthy eating and lack time, skills and equipment to prepare and cook food at home	**Then** this can lead to a greater frequency of consumption of HFSS processed foods increasing the risk of diet‐related ill health	**Because** of confusion about how/why to make healthy choices, competing priorities, e.g., belief that immediate priority is to avoid hunger and provide foods that are filling and acceptable rather than healthy, perception that take away foods are more cost‐effective and attractive alternatives to cooking at home and lack of confidence to cook

### 3.4 Understanding Community Needs

Four consolidated CMOCs were identified for understanding community needs (Table 3). Complex social and cultural factors were found to negatively impact people's decision‐making and food choices [47, 48, 51, 57, 58, 59, 60, 63, 66, 69, 72, 76]. This includes structural issues, such as a lack of transport or a decline in local shops [72]. If organisations work collaboratively and engage with vulnerable groups to understand these complexities then CNIs are more likely to meet identified needs and improve nutrition. This is because people feel their knowledge, beliefs and priorities are acknowledged and more tailored, practical, trusted advice and support are provided (CMOC 1) [47, 48, 51, 58, 59, 69]. Where there is a failure to understand community needs e.g., providing healthy eating information without engaging with the impact of socioeconomic deprivation on food decisions, then the mechanisms (feeling acknowledged, and tailored, practical, trusted support) are absent and interventions are likely to have a limited impact (CMOC 2) [48, 63, 72].

Building partnerships and trusting relationships was an important mechanism that facilitated an understanding of community needs resulting in positive outcomes (CMOC 3) [57, 59, 60, 63, 69, 76]. For example, staff delivering community cooking courses for people with low literacy levels encouraged group discussion and interaction, which instilled confidence and trust in participants to share their life experiences and acquire new skills [57]. This increased their self‐value, cooking self‐efficacy and general confidence, and facilitated positive dietary change. Notably, HCPs recognised that young women in their care often experience turbulent lives and relationships of trust needed to be established before healthy lifestyle advice could be prioritised [63]. Furthermore, facilitators of a healthy lifestyle programme for diverse cultural groups reported that building relationships with community members ensured the programme's content and approach was culturally appropriate and maximised engagement [76]. Similarly, youth workers reported young people's increased resilience and ability to mitigate the negative impact of food poverty when allowed to express and articulate wider social and political influences [60].

HCPs capability to effectively and inclusively meet people's health needs and facilitate dietary behaviour change was contingent upon several mechanisms. These were critical reflections on their own emotions, attitudes and beliefs concerning weight and shape, and gaining knowledge and understanding of social and cultural factors that impact food decisions (CMOC 4), nutrition, behaviour change skills and confidence to provide healthy lifestyle support. [47, 48, 58, 63, 66]. These mechanisms can be activated by improving understanding (through education and training) of complex social and cultural determinants that can impact diet and by addressing expressed prejudices related to stereotypes [47, 63, 66]. In addition, training can improve HCPs' skills in motivational interviewing, how to have healthy conversations and goal setting [66] (in addition, see CMOC 9). If HCPs lack the belief that they can make a difference to women's behaviour during pregnancy and do not understand complex social and cultural factors that impact nutrition then these mechanisms (critical reflection and acquiring knowledge, skills and confidence) are unlikely to activate.

### 3.5 Consistent Nutrition Messages

Four consolidated CMOCs were identified for consistent nutrition messages (Table 4). Pregnant women, first‐time parents and older adults were at greater risk of low adherence to dietary recommendations and poor nutritional intake [39, 40, 42, 59, 74]. This was despite a high desire to provide a healthy diet for themselves and/or their family. Mechanisms contributing to this outcome were confusion and mistaken beliefs about how and why to make healthy food choices and commonly held perceptions such as ‘eat for two’ during pregnancy (CMOC 5) [39, 42, 59]. A further mechanism was information seeking, often from informal sources such as the internet or from family members. This can result in inadvertently following inappropriate advice e.g., inappropriate early introduction to solid foods for infants or including foods high in fat, sugar and salt (HFSS) in children's diet [42, 74]. Incomplete knowledge of nutrition and poor access to reliable nutrition information and advice from trusted sources were prominent contextual factors.

HCPs play a pivotal role in providing consistent nutrition messages, minimising confusion from conflicting information and addressing people's knowledge‐attitude gaps [40, 58, 59]. In congruence with CMOC1 and CMOC2, when HCPs had knowledge and understanding of people's nutritional needs (as well as wider social determinants) and could adapt the advice they provided to meet these needs, this facilitated positive nutrition outcomes. Mechanisms here were people felt supported when correct nutrition information was available and confusion from conflicting messages was avoided (CMOC 6) [58, 59]. For older adults, carers' nutrition knowledge and awareness of increased risks of poor appetite and nutritional deficiency amongst this population group, prevented unsafe practice. This was because there was less likelihood of older adults being advised to following restricting diet that were potentially harmful [59]. If people are not able to access reliable, trusted, correct nutrition information and advice adapted to their needs (tailored) then the mechanisms identified in CMOC 5 will prevail.

Evaluation of CNIs such as school holiday schemes [56] and healthier catering awards [64] found that unhealthy food was often provided and they were more affordable than healthy options. This could undermine efforts to encourage healthy food consumption by conveying conflicting messages to the public [56, 64, 73]. In this situation, confusion due to food provision not aligning with healthy eating recommendations, unhealthy foods perceived as ‘treats’, and the perception that healthy foods are less affordable were the main mechanisms. Examples of ways to avoid activating these mechanisms (confusion, misalignment of messages) included young people cooking healthy food alongside staff during healthy cooking sessions in schools and efforts to increase the affordability of basic, healthy ingredients including fruit and vegetables [56, 73].

Learning about nutrition, health, food preparation skills, food labels, portion sizes and encouraging children to eat well were notable mechanisms that facilitated dietary behaviour change [42, 49, 52, 55, 57, 61, 71, 76]. Other outcomes were adopting healthy cooking habits into daily routines, managing food waste and budgeting [55, 61]. CNIs that provided nutrition education and practical cooking skills development opportunities activated these mechanisms. Particular attention should be paid to utilising different communication channels [52, 61] and targeting life stages when people are more receptive (early parenthood, young adults) [42, 71]. For young adults in particular, this approach provided the tools needed for a healthy lifelong relationship with food and a greater capacity for cooking at home [71].

### 3.6 Knowledgeable, Skilled, Confident Practitioners/Facilitators

Four consolidated CMOCs were identified for knowledgeable, skilled, confident practitioners/facilitators (Table 5). HCPs can influence people's health behaviours through brief healthy lifestyle interventions and conversations. HCPs who are confident in their nutrition knowledge and have self‐efficacy in their ability to sensitively raise topics such as weight gain during pregnancy or family eating patterns are more motivated to discuss health behaviours (CMOC 9) [40, 50, 63, 66, 81]. Furthermore, increased confidence and self‐efficacy can alleviate anxiety and fear of coming across as judgemental, lacking specialist knowledge or causing offence or upset, and damaging their relationships with women or parents (CMOC 10) [63, 66, 81]. These mechanisms lead to an increased likelihood that dietary advice and support will be provided and ultimately people receiving the information feel supported to modify their behaviour [50]. Training and education in nutrition, and behaviour change techniques such as motivational interviewing and goal setting can activate these mechanisms. HCPs who lack confidence may avoid raising sensitive topics as in the absence of confidence and self‐efficacy, the dominant mechanisms are anxiety and fear of causing upset (CMOC 10).

During pregnancy and early parenthood, dietary behaviour change was more likely to be facilitated if the advice and support were regarded as reliable and trustworthy and focused on building people's confidence and self‐efficacy to set incremental goals (CMOC 11) [39, 40, 50, 74]. Other notable mechanisms were a sense of ownership over the goals, accomplishment and pride when they were met [50]. Mechanisms were contingent upon HCPs having knowledge, skills and confidence to provide positive, practical nutrition messages and encourage people to set goals they were able to achieve [40, 50]. Without this support, incomplete knowledge and mistaken beliefs about nutrition could remain active mechanisms (CMOC 5).

Nutritional care for older people in care settings was optimised when carers had good knowledge and understanding (through training) of the specific nutritional needs of older adults and were alert to signs of undernutrition (CMOC 12) [53, 70]. This enabled them to advise and support people and their relatives and to adapt information to individual needs. Notable mechanisms, addressing mistaken beliefs such as ‘being thin is healthy’ and confusion over conflicting messages are avoided as previously described (CMOC 5).

### 3.7 Practising New Skills

Five consolidated CMOCs were identified for practising new skills (Table 6). There were positive outcomes for CNIs that provided opportunities for people to socialise with others and practise food preparation skills [44, 48, 55, 68, 73]. Outcomes included less food waste [55, 67], reduced time and money spent preparing and cooking food, increased cooking from raw ingredients, reduced intake of HFSS convenience/fast foods, healthier food choices and better‐quality diets [44, 48, 55, 68, 73]. Dominant mechanisms that were activated included increased knowledge and confidence of how to cook in bulk, store and freeze foods properly and use leftovers to make another meal (CMOC 13). In addition, enjoyment and self‐efficacy to cook home‐prepared meals were active mechanisms, particularly for groups most likely to rely on takeaway foods (e.g., working overtime, lower household income and younger people). Healthy eating at the family level was improved by including adults responsible for food provisioning in the home in school‐based practical cooking skills activities. Learning to shop more thriftily, bulk buy, batch cook and adjust recipes to meet family preferences were skills that led to greater self‐efficacy to prepare and cook healthy meals (CMOC 14) [44, 68, 79]. For children and young people, opportunities to practise food preparation skills activated mechanisms such as greater food and health motivation and cooking identity, supporting better quality outcomes that track into adult life. Opportunities to practise skills and participate in fun activities can ignite enjoyment and pleasure from cooking and reduce stress when involving young children (CMOC 15) [48, 51, 67].

Having the opportunity to experiment and develop a repertoire of well‐known dishes increases the likelihood of cooking from scratch for parents on a low income [44, 48, 68]. This is because it helps to eliminate the fear of wasting food and money instilled by previous personal disasters in the kitchen [44]. Families on a low income may not have a safe space for experimenting and learning about food preparation and cooking. In these circumstances, fear of failure or that food will be rejected by family members when served are the prominent mechanisms [48]. In this case, parents are less likely to repeatedly experiment to develop new skills to prepare minimally processed foods and children miss opportunities to try new foods (CMOC 16) [44, 48, 68].

Some circumstances resulted in increased consumption of HFSS foods that could increase people's risk of diet‐related ill health. These circumstances reflect the complex factors that impact people's food decisions, as evidenced for CMOCs 1–4, such as lack of knowledge of what constitutes healthy eating [42], and lack of time [47, 54], skills and equipment [43, 73] to prepare and cook food at home. Competing priorities were evident such as parents' belief that the immediate priority when feeding their children is to avoid hunger and provide foods that are filling and acceptable rather than healthy [48]. Other potential causative mechanisms are a lack of confidence and self‐efficacy to cook (CMOC 13) and the perception that takeaway foods are a more cost‐effective and attractive alternative to cooking at home (CMOC 17) [73].

**Figure 4:**
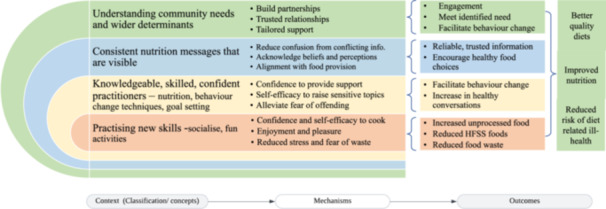
Initial programme theory.

Our IPT is shown in Figure 4. The full programme theory is summarised in Supporting Information: File 6.

## 4 Discussion

The first objective of this review was to begin to determine the underpinning IPT for how CNIs like NSFL in Wales work in supporting people, particularly from SED communities, to access a healthy diet.

### 4.1 How Do CNIs Support People, Particularly Those From SED Communities, to Access a Healthy Diet?

Findings from this review confirmed the importance of all stakeholders involved in CNIs understanding the complex structural and sociocultural factors that can make it more difficult for people from SED communities to access and consume a healthy diet than less disadvantaged communities [8, 87, 88, 89]. Acknowledging these unequal circumstances and flexibly tailoring interventions to address identified barriers were important contextual factors for supporting dietary behaviour change. Failure to acknowledge the physical and social environment when trying to understand behaviour creates ‘the fundamental attribution error’ meaning, a general tendency to blame individuals for something that is out of their control [90]. Involving community members as equal partners in CNI development and implementation through community participation and engagement can ensure CNIs support health improvement and reduce inequalities in health [91].

Incomplete nutrition knowledge of both those providing and those receiving nutrition information was identified in this review and has been reported in other studies [92, 93, 94]. Misinformation is widespread in the food domain, particularly among people or groups whose ability to access and understand health information, referred to as health literacy, is low [95]. Lower levels of trust in nutrition information among people from lower SED groups have also been reported [95]. This may, in part, be due to the prevalence and spread of health misinformation on online platforms, which has become a significant problem in recent years [92] and to competing messages inferred implicitly via unhealthy food environments [93]. HCPs are a trusted source of advice and information and missed opportunities to communicate reliable, easy‐to‐understand nutrition information was concerning, given the widespread confusion that exists [92, 93, 94, 96].

This review offers useful insights into the potential causal mechanisms whereby consistent nutrition messages, a component of nutrition education [96], can influence dietary behaviour change, particularly for SED communities, when combined with other contextual factors. For example, frontline workers are in a prime position to communicate consistent nutrition messages through their everyday contact with the public. Our findings demonstrated that confident, knowledgeable HCPs skilled in using behaviour change techniques (BCTs), such as goal setting, were more likely to discuss eating habits during their conversations with the public. This was because they had greater self‐efficacy in their ability to provide person‐centred support and were less fearful of appearing judgmental. For those receiving the support, this triggered confidence, motivation and self‐efficacy to set goals for behaviour change and pride when goals were met. Regarding CNIs delivered by facilitators who used active or experiential learning methods, such as group discussions and interaction, engagement increased for people with low literacy levels. This was because trusting, respectful relationships developed between participants, who were willing to share their life experiences with others. Participants also developed an understanding of the nutritional value of food and self‐efficacy to make dietary changes. These findings concur with existing best practice principles that suggest CNIs with a behavioural theory basis are more effective than information or knowledge‐based interventions [21]. In addition, for low literacy and low‐income groups, experiential education, e.g., cooking, food preparation, shopping tours and other hands‐on activities, is preferable to didactic or lecture‐style interventions [18, 21].

Upskilling the wider health and community‐based workforce in nutrition is one of the most common capacity‐building strategies used in public health nutrition practice to enhance the reach and effectiveness of CNIs [97, 98, 99]. Indeed, this is the main objective of the NSFL programme in Wales [17, 35]. Findings from this review demonstrate the important context that training provides [99]. Training should integrate the complex social determinants and structural factors that impact people's diets, particularly in SED communities, along with BCTs, person‐centred approaches and adult learning theory, in addition to nutrition. This can boost staff self‐confidence in nutrition knowledge, enhance their self‐efficacy in using BCTs and alleviate fears of causing offence or providing misinformation when supporting others in making dietary changes [37, 46, 60, 63, 78]. This concurs with Begley [100], as cited by Vidgen [21], that CNIs should build participants' self‐efficacy, confidence, self‐motivation and perceived control over their eating. Therefore, it is important that programmes include some form of intention formation activity, for example, goal setting, provide feedback on skill development and review behavioural goals [21, 101].

Finally, CNIs create physical and social opportunities for people to meet with others from their community who are in similar circumstances to themselves to practise new food‐related skills and develop food literacy. Food literacy is an evolving term for ‘a collection of inter‐related knowledge, skills and behaviours required to plan, manage, select, prepare and eat food to meet needs’ [21]. There was a lack of reference to food literacy within the reviewed documents. A dearth of research on this topic conducted in the UK has been identified elsewhere [102] and the UK lags behind international developments in this comprehensive approach to food education, particularly for our future generations [103]. Nevertheless, food skills programmes included in this review produced a range of positive outcomes, for example, reduced reliance on HFSS foods and improved diet quality [44, 55, 57, 68]. These findings were consistent with other research, which utilised a realist approach to investigate cooking skills programme theory [104, 105]. Maugeri et al. [105] found that if nutrition education cooking interventions with SED populations included hands‐on cooking and a skilled facilitator, this produced a range of positive outcomes e.g., increase in knowledge, cooking skills and dietary change. This was because of mechanisms, such as individual self‐efficacy, knowledge gain, family support and an expectation of positive health outcomes, for example, self‐management of type 2 diabetes. Similarly, Blamey et al. [104] identified social interaction, motivation, self‐efficacy, confidence and self‐belief, and social support as important mechanisms for dietary improvement. Other potential programme theories that were congruent with the findings in this review were opportunities to experiment with new foods without fear of waste or rejection by family members [104, 105].

### 4.2 Specific Programme Theory That May Warrant Further Review

The second objective of this review was to identify a specific programme theory that may warrant a full realist review, or realist evaluation, to test and refine the theory and inform the future direction of the NSFL programme in Wales. A quarter of the reviewed documents (*n* = 12) mentioned underpinning theory; however, the detail of how the constructs of the relevant theory were applied was generally weak. Further research is needed to fully explore the substantive social and psychological theory that could inform a more precise programme theory for NSFL [83, 106, 107] (e.g., see Supporting Information: File 7). Therefore, findings from this scoping review should be considered a first step towards developing the programme theory for how NSFL works.

The consolidated CMOCs (IPT) for understanding community needs; consistent nutrition messages; knowledgeable, skilled, confident practitioners and practicing new skills, provided valuable insight into potential mechanisms (building partnerships and trusting relationships, reducing confusion from conflicting messages, practitioners' confidence and self‐efficacy to provide support and individuals' confidence and self‐efficacy to feed themselves and their family well) that could positively impact diet quality and health outcomes. The contingent contextual factors uncovered in this review need further discussion with stakeholders. Therefore, realist evaluation to test, refute and iteratively refine the IPT into a final programme theory with programme stakeholders is needed.

Environmental, social and cultural factors that influence people's diets should also be considered when testing the IPT [86]. This would determine the full contribution NSFL makes towards the required cultural shift in food‐related social norms and practices and food system transformation that is needed to protect and promote population health [88, 89]. This could contribute to the development of a portable theory that can usefully inform other public health promotion programmes in the future.

### 4.3 Implications for Implementing and Evaluating CNIs Such as NSFL

This review of how the NSFL programme might work has generated new insights and learning, which can usefully inform and guide equitable service delivery across all areas of Wales. In summary, these considerations include: fully understanding the complex determinants that impact access and affordability of healthy food; involving local people at all stages of intervention development; providing nutrition education and training for health and care professionals; providing consistent, evidence‐based nutrition messages; enabling people to set incremental, realistic goals for dietary behaviour change and providing opportunities to practise food preparation skills (see Box 1).

Box 1. **Specific considerations for equitable service development and evaluation**

1. Fully understand and acknowledge the complex social determinants and structural factors that impact people's diets, including the food environment and, in partnership with communities and organisations, prioritise equitable access, availability, affordability and acceptability of healthy and sustainable food for everyone (CMOC 1–4 and 7).
2. Involve people at all stages of intervention development through coproduction, to identify barriers (financial constraints, equipment, dining facilities), increase engagement, ensure provision is culturally appropriate and to meet identified needs (CMOC 3).
3. Provide opportunities for people to socialise, build relationships, share meals in community venues and to learn new skills e.g., meal planning, preparation, cooking, storing and freezing foods and using leftovers (CMOC 13–16).
4. Provide education and training for health and care professionals and other community workers in trusted positions who can influence population dietary behaviour change. This will build workforce capacity to diffuse evidence‐based nutrition messages and behaviour change strategies via existing channels and organisational structures e.g. Healthy Child Wales [108] (CMOC 4,9,10,12).
5. Provide consistent, evidence‐based, easy‐to‐understand, practical food and nutrition messages through all food‐related activities and interventions and ensure food provision in settings aligns with population dietary guidelines (CMOC 5– 8).
6. Enable people to set their own incremental, achievable goals to increase self‐efficacy and motivation to make dietary behaviour changes (CMOC 11).
7. Provide opportunities to repeatedly practise meal planning and preparation skills to develop capability and self‐efficacy to prepare healthy meals from basic ingredients, form new habits and increase motivation and the likelihood of lasting behaviour change (CMOC 13–17).

### 4.4 Strengths and Limitations

The main strength of this review is that to our knowledge, a programme theory for what works, for whom and under what circumstances for CNIs in SED communities, has not been determined in the UK. This review, therefore, provides new insights that may be of interest to others working in public health nutrition and dietetics. The lack of a development framework for conducting a scoping review that incorporates realist principles was a limitation as although this approach may be considered novel, it required considerable reflection and interpretation of existing guidance for scoping reviews and realist reviews to decide an appropriate methodology. Restricting sources of information to the UK may have limited the potential to uncover other causal mechanisms from international literature, for example, in the fields of culinary nutrition (USA) or food literacy (Australia). A further limitation was the challenge involved in extracting and interpreting the multiple causal mechanisms and pathways reflected in the consolidated CMOCs described in this review. Forty‐five sources of evidence yielded 170 tentative CMOCs, which demonstrates the complexity of the issues addressed. Identifying which elements of programme theory were context, or mechanism required discussion with expert reviewers to substantiate and refine. Nevertheless, the review has produced valuable findings that have the potential to inform the future direction of CNIs such as those supported by the NSFL programme in Wales.

## 5 Conclusion and Recommendations

This review yielded evidence that informed the development of an IPT for how CNIs can support people in more SED areas to access a healthy diet. Interventions ‘worked’ when they acknowledged and addressed identified barriers to healthy eating, provided reliable, trusted, easy‐to‐understand nutrition messages, were delivered by confident, knowledgeable practitioners, and facilitated strategies such as meal preparation. Reactions generated included feeling supported and understood, trust in the information and support provided, practitioners' confidence and self ‐efficacy to provide tailored support and public confidence and self‐efficacy to achieve goals and prepare nutritious, affordable meals. The findings from this review provide valuable insights and learning that can usefully inform the current and future delivery of CNIs such as NSFL in Wales. Realist evaluation is recommended, to test and refine the IPT developed from this review. Integrating a Social Return on Investment (SROI) analysis would provide evidence of the programme's social as well as economic value and impact. This would deepen our understanding of how CNIs work, particularly for SED population groups to promote optimum nutrition and reduce diet‐related health inequalities.

## Author Contributions

J. Lisa Williams prepared the initial protocol, undertook the searches, document screening by title, abstract and full text, coding of CMOCs, developed the initial programme theory and prepared the manuscript. Carolyn Wallace undertook duplicate screening of abstracts, provided expertise and support for the development of CMOCs, reviewed and commented on the final manuscript. Teresa Filipponi undertook duplicate screening of titles and provided expertise and support for writing the final manuscript.

## Conflicts of Interest

J. Lisa Williams is the project co‐ordinator and coauthor of nutrition training materials for the all Wales nutrition education programme Nutrition Skills for Life. All steps of the scoping review process were clearly documented and verified by project reviewers to avoid any bias.

## Supporting information

Supplementary file 1. RAMESES and PRISMA‐ScR checklists.

Supplementary file 2. PCC table, search terms, eligibility criteria, data extraction tool.

Supplementary file 3. Document quality.

Supplementary file 4. Descriptive characteristics of included documents.

Supplementary file 5. Data for consolidated CMOCs.

Supplementary file 6. Overview of the Nutrition Skills for Life Initial Programme Theory.

Supplementary file 7. Relevant substantive theory.

## Data Availability

Data sharing is not applicable to this article as no new data were created or analysed in this study.
