# Supplementary material for: A Realist Scoping Review of Community Nutrition Interventions in the UK: Implications for the ‘Nutrition Skills for Life’ Programme
Source: J Hum Nutr Diet. 2025 Jan 8;38(1):e70008. doi: 10.1111/jhn.70008 (PMC11707723; doi:10.1111/jhn.70008)
Supplement: Supplementary file 1 — RAMESES and PRISMA‐ScR checklists. [file JHN-38-0-s003.docx]

Supplementary file 1. RAMESES and PRISMA-ScR checklists

RAMESES checklist

| Reporting item | | Description of item | Reported on page(s) |
| --- | --- | --- | --- |
| TITLE | | | |
| 1 |  | In the title, identify the document is a realist synthesis or review | Title page |
| ABSTRACT | | | |
| 2 |  | While acknowledging publication requirements and house style, abstracts should ideally contain brief details of: the study’s background, review question or objectives; search strategy; methods of selection, appraisal, analysis and synthesis of sources; main results; and implications for practice. | Page 1 |
| INTRODUCTION | | | |
| 3 | **Rationale for review** | Explain why the review is needed and what it is likely to contribute to existing understanding of the topic area. | Pages 3-5 |
| 4 | **Objectives and focus of review** | State the objective(s) of the review/or the review question(s). Define and provide a rationale for the focus of the review | Pages 3-5 |
| METHODS | | | |
| 5 | **Change in the review process** | Any changes made to the review process that was initially planned should be briefly described and justified. | Page 6 |
| 6 | **Rationale for using realist synthesis** | Explain why realist synthesis was considered the most appropriate method to use. | Page 6 |
| 7 | **Scoping the literature** | Describe and justify the initial process of exploratory scoping of the literature. | Pages 6 and 7 |
| 8 | **Searching processes** | While considering specific requirements of the journal or other publication outlet, state and provide a rationale for how the iterative search was done. Provide details on all sources accessed for information in the review. Where searching in electronic databases has taken place, the details should include, for example, name of the database, search terms, dates of coverage and date last searched. If individuals familiar with the relevant literature and/or topic area were contacted, indicate how they were identified and selected. | Pages 6 and 7  Supplementary file 2 |
| 9 | **Selection and appraisal of documents** | Explain how judgements were made about including and excluding data from documents, and justify these. | Pages 6 and 7  Supplementary file 2 |
| 10 | **Data extraction** | Describe and explain which data or information were extracted from the included documents and justify this selection. | Page 7  Supplementary files 2 & 3 |
| 11 | **Analysis and synthesis processes** | Describe the analysis and synthesis processes in detail. This section should include information on the constructs analysed and describe the analytic process. | Page 7 |
| RESULTS | | | |
| 12 | **Document flow diagram** | Provide details on the number of documents assessed for eligibility and included in the review with reasons for exclusion at each stage as well as indication of their source of origin (for example, from searching databases, reference lists and so on). You may consider using the example templates (which are likely to need modification to suit the data) that are provided. | Page 8 Figure 1 |
| 13 | **Document characteristics** | Provide information on the characteristics of the documents included in the review. | Pages 9 - 10  Supplementary file 3 |
| 14 | **Main findings** | Present the key findings with a specific focus on theory building and testing. | Pages 10-18 |
| DISCUSSION | | | |
| 15 | **Summary of findings** | Summarise the main findings, taking into account the review’s (objectives), research question(s), focus and intended audience(s). | Pages 19-23 |
| 16 | **Strengths, limitations and future research directions** | Discuss both the strengths of the review and its limitations. These should include (but need not be restricted to) (a)consideration of all the steps in the review process and (b) comment on the overall strength of the evidence supporting the explanatory insights which emerged. The limitations identified may point to areas where further work is needed. | Pages 23-24 |
| 17 | **Comparison with existing literature** | Where applicable, compare and contrast the review’s findings with the existing literature (for example, other reviews) on the same topic. | Pages 19-22 |
| 18 | **Conclusion and recommendations** | List the main implications of the findings and place these in the context of other relevant literature. If appropriate, offer recommendations for policy and practice. | Pages 22-24 |
| 19 | **Funding** | Provide details of funding source (if any) for the review, the role played by the funder (if any) and any conflicts of interest of the reviewers. | Title page |

PRISMA ScR checklist

| **SECTION** | **ITEM** | **PRISMA-ScR CHECKLIST ITEM** | **REPORTED ON PAGE #** |
| --- | --- | --- | --- |
| **TITLE** | | | |
| Title | 1 | Identify the report as a scoping review. | Title page |
| **ABSTRACT** | | | |
| Structured summary | 2 | Provide a structured summary that includes (as applicable): background, objectives, eligibility criteria, sources of evidence, charting methods, results, and conclusions that relate to the review questions and objectives. | Page 1 |
| **INTRODUCTION** | | | |
| Rationale | 3 | Describe the rationale for the review in the context of what is already known. Explain why the review questions/objectives lend themselves to a scoping review approach. | Pages 3-5 |
| Objectives | 4 | Provide an explicit statement of the questions and objectives being addressed with reference to their key elements (e.g., population or participants, concepts, and context) or other relevant key elements used to conceptualize the review questions and/or objectives. | Pages 3-5  Supplementary file 2 |
| **METHODS** | | | |
| Protocol and registration | 5 | Indicate whether a review protocol exists; state if and where it can be accessed (e.g., a Web address); and if available, provide registration information, including the registration number. | Page 6 |
| Eligibility criteria | 6 | Specify characteristics of the sources of evidence used as eligibility criteria (e.g., years considered, language, and publication status), and provide a rationale. | Page 6  Supplementary file 2 |
| Information sources* | 7 | Describe all information sources in the search (e.g., databases with dates of coverage and contact with authors to identify additional sources), as well as the date the most recent search was executed. | Page 6  Supplementary file 2 |
| Search | 8 | Present the full electronic search strategy for at least 1 database, including any limits used, such that it could be repeated. | Supplementary file 2 |
| Selection of sources of evidence† | 9 | State the process for selecting sources of evidence (i.e., screening and eligibility) included in the scoping review. | Pages 6-7  Supplementary file 2 |
| Data charting process‡ | 10 | Describe the methods of charting data from the included sources of evidence (e.g., calibrated forms or forms that have been tested by the team before their use, and whether data charting was done independently or in duplicate) and any processes for obtaining and confirming data from investigators. | Page 7 |
| Data items | 11 | List and define all variables for which data were sought and any assumptions and simplifications made. | Supplementary file 2 |
| Critical appraisal of individual sources of evidence§ | 12 | If done, provide a rationale for conducting a critical appraisal of included sources of evidence; describe the methods used and how this information was used in any data synthesis (if appropriate). | Page 8  Supplementary file 3 |
| Synthesis of results | 13 | Describe the methods of handling and summarizing the data that were charted. | Page 8 |
| **RESULTS** | | | |
| Selection of sources of evidence | 14 | Give numbers of sources of evidence screened, assessed for eligibility, and included in the review, with reasons for exclusions at each stage, ideally using a flow diagram. | Page 8 Figure 1 |
| Characteristics of sources of evidence | 15 | For each source of evidence, present characteristics for which data were charted and provide the citations. | Pages 9-10  Supplementary file 4 |
| Critical appraisal within sources of evidence | 16 | If done, present data on critical appraisal of included sources of evidence (see item 12). | Page 8  Supplementary file 3 |
| Results of individual sources of evidence | 17 | For each included source of evidence, present the relevant data that were charted that relate to the review questions and objectives. | Supplementary file 4 |
| Synthesis of results | 18 | Summarize and/or present the charting results as they relate to the review questions and objectives. | Pages 10-18 Supplementary files 4 & 5 |
| **DISCUSSION** | | | |
| Summary of evidence | 19 | Summarize the main results (including an overview of concepts, themes, and types of evidence available), link to the review questions and objectives, and consider the relevance to key groups. | Pages 19-22 |
| Limitations | 20 | Discuss the limitations of the scoping review process. | Pages 23-24 |
| Conclusions | 21 | Provide a general interpretation of the results with respect to the review questions and objectives, as well as potential implications and/or next steps. | Pages 19-24 |
| **FUNDING** | | | |
| Funding | 22 | Describe sources of funding for the included sources of evidence, as well as sources of funding for the scoping review. Describe the role of the funders of the scoping review. | Title page |
