# Supplementary material for: A Realist Scoping Review of Community Nutrition Interventions in the UK: Implications for the ‘Nutrition Skills for Life’ Programme
Source: J Hum Nutr Diet. 2025 Jan 8;38(1):e70008. doi: 10.1111/jhn.70008 (PMC11707723; doi:10.1111/jhn.70008)
Supplement: Supplementary file 2 — PCC table, search terms, eligibility criteria, data extraction tool. [file JHN-38-0-s002.docx]

**Supplementary file 2. PCC table, search terms, eligibility criteria, data extraction tool**

**PCC table**

| **Participants** | **Concept** | **Context** |
| --- | --- | --- |
| Adults 19+ | Community nutrition interventions | UK |
| Parents/families | Dietary behaviour | Community based |
| No comorbidities | Health/ dietary behaviour change | Reducing health inequalities |
| No specific dietary needs | Food / nutrition literacy | Reducing use of services |
|  | Food /nutrition skills | Prevention of ill health |
|  | Knowledge/attitudes/practices |  |
|  | Food preparation/cooking skills |  |
|  | Social value/ reduce social isolation |  |
|  | Increased resilience/coping strategies |  |
|  | Barrier and facilitators to behaviour change |  |
|  | Food access/ food security |  |
|  | Sustainable diets |  |

**Search terms**

**Medline (Ovid)**

exp Diet/ **OR** exp Nutrition Therapy/ **OR** exp Food/ **OR** exp Diet, Healthy/ **OR** (Diet* or food or feed* or nutrition*).mp. [mp=title, abstract, original title, name of substance word, subject heading word, floating sub-heading word, keyword heading word, organism supplementary concept word, protocol supplementary concept word, rare disease supplementary concept word, unique identifier, synonyms]

**AND**

exp Community Health Services/ **OR** exp Community Health Workers/ **OR** ((communit* or home or rural) adj3 (service* or practice or health or intervention)).mp. [mp=title, abstract, original title, name of substance word, subject heading word, floating sub-heading word, keyword heading word, organism supplementary concept word, protocol supplementary concept word, rare disease supplementary concept word, unique identifier, synonyms]

**AND**

exp Health Behavior/ **OR** ((diet* or nutrition* or food or feed*) adj3 (behaviour or behavior* or habit* or intake)).mp. [mp=title, abstract, original title, name of substance word, subject heading word, floating sub-heading word, keyword heading word, organism supplementary concept word, protocol supplementary concept word, rare disease supplementary concept word, unique identifier, synonyms] **OR** exp Feeding Behavior/ **OR**  (health adj3 (behaviour* or behavior* or habit*)).mp. [mp=title, abstract, original title, name of substance word, subject heading word, floating sub-heading word, keyword heading word, organism supplementary concept word, protocol supplementary concept word, rare disease supplementary concept word, unique identifier, synonyms] **OR** (health adj3 (behaviour* or behavior* or habit*)).tw. **OR** ((diet* or nutrition* or food or feed*) adj3 (behaviour or behavior* or habit* or intake)).tw.

**AND**

exp Cooking/ **OR** ((food or nutrition or cooking) adj3 (literacy or skill* or knowledge)).tw. **OR** exp Health Knowledge, Attitudes, Practice/ **OR** Health Education/ or exp Health Knowledge, Attitudes, Practice/

**Eligibility criteria**

| **Inclusion** | **Exclusion** |
| --- | --- |
| Adults 19+ | International- non-UK |
| Parents | Secondary healthcare |
| Families | Tertiary healthcare |
| Community based | Treatment of disease -specific therapeutic diets e.g., Coeliac Disease, Diabetes |
| Community settings e.g., schools/nurseries with family involvement | Hospital in-patients |
| Nutrition training | Children |
| Nutrition education | Adults with co morbidities e.g., obesity, cardiovascular disease, hypertension |
| Community health workers/lay workers/lay advisers | Community settings e.g., schools, nurseries without family involvement |
| Nutrition literacy/food literacy | Prior to January 2012 |
| Nutrition/food/food preparation/cooking skills |  |
| Nutrition/food/diet intervention or project |  |
| Food access |  |
| Food security |  |
| Health behaviour change |  |
| Dietary behaviour change |  |
| Dietary habits |  |
| Reduced risk of diet related illness |  |
| Reducing health inequalities/reducing food insecurity |  |
| Reducing burden/use of services |  |
| Social value/ reduce social isolation |  |
| Increased resilience/coping strategies |  |
| UK |  |
| 2012-2022 |  |
| English language |  |

**Data extraction tool**

| Title | Full title of paper |
| --- | --- |
| Authors | List all authors |
| Date of publication | Enter as actual date or if month and year list as 1^st^ of month |
| Where in the UK | Where study conducted and whether socio demographic factors listed |
| Aims/purpose of study | From main aim, determine what factors are being explored that might be key propositions in CMOCs |
| Research or evaluation design approach | Mixed method/Qual/Quant/review for ease of description. |
| Study methodology | Methodology e.g., phenomenological, action research. MM with concurrent/parallel and at which point is it integrated. |
| Study methods | Survey/interviews/focus groups |
| Sampling plan outlined | Yes/No |
| Sampling strategy | What sampling strategy used? |
| Participants/ details/ age/sex/number | Record the demographics of the study population. This could contribute valuable information as to whether IPT is different for different life stages e.g., pregnancy, older adults |
| Is the intervention intended for individuals/ settings /communities? | This will allow for sorting interventions by level and to determine differences in mechanism at indiv/place/environmental level e.g., resource/ reasoning.  Further note on context and variation in response to intervention  ***If subject is individual****, the programme through all its phases may be met with variation in, entrenchment of habits, level of risk, capacity for learning*  ***If the subject is a locality,*** *limiting factors may be the extent of internal division, availability of existing resources, or the level of community action.* |
| Provide further detail about setting/ community involved if relevant | Setting involved so similar settings/papers grouped together |
| Who delivers the intervention / Mode of delivery Description/ content/ duration of the intervention | Is this qualified nutrition professionals (state details), lay workers, what training do deliverers have, is this specified? State if not specified. Where is it delivered? E.g., train the trainer, delivery manual, refresher training etc. 2 hours per week for 10-12 weeks. Online v face-to-face To contribute towards identifying CMO-Cs, particularly **mechanism of change.** Programme dose, self-efficacy, agency, |
| Theoretical basis / substantive theory /BCT mentioned? | Record if mentioned and what specific factors e.g., social cognitive theory (learning by observation/modelling). To contribute towards determining IPT in preparation for testing and refining, and/or behaviour change technique |
| **Outcomes** | Document the main outcomes of the study. Dietary change, what specific dietary changes were made. What other food/nutrition related outcomes were documented. How might this work so that SED groups are supported to access good food/eat better? **How does the intervention work/ not work?** |
| **Mechanisms** | Document **why the intervention works/ doesn’t work** and if this is specified particularly for LSE groups. What are the resources/lack of resources or reasoning (micro) behind why the intervention works/doesn’t work? |
| **Context** | Document the wider context, the factors that need to be in place for the mechanisms to be ‘triggered’ or ‘fired’ (gunpowder analogy) wider embeddedness, culture, social norms and infrastructure. |
| **Impact** | Is longer term impact mentioned e.g., food system change/ obesogenic environment |
