# Supplementary material for: A Realist Scoping Review of Community Nutrition Interventions in the UK: Implications for the ‘Nutrition Skills for Life’ Programme
Source: J Hum Nutr Diet. 2025 Jan 8;38(1):e70008. doi: 10.1111/jhn.70008 (PMC11707723; doi:10.1111/jhn.70008)
Supplement: Supplementary file 3 — Document quality. [file JHN-38-0-s001.docx]

**Supplementary file 3.** Data sources for CMOCs contributing to the IPT.

Synthesis of all documents yielded 170 CMOCs. Ten broad classifications for the initial CMOCs were identified.

Four main classifications with the highest number of contributing CMOCs were identified (as highlighted below) for further analysis

| **Paper** | **1. Food environment** | **2. Mitigating food poverty** | **3. Understanding community need** | **4. Social meaning of food** | **5. Consistent nutrition messages- Inc. Digital (D)** | **6. Building strong support networks** | **7. Active learning** | **8. Knowledgeable, skilled, confident practitioners/facilitators** | **9 .Goal Setting** | **10. Practicing new skills** | **RAG** |
| --- | --- | --- | --- | --- | --- | --- | --- | --- | --- | --- | --- |
| 1 ^(38)^ |  |  |  | **🗸** |  |  |  |  |  |  |  |
| 2 ^(39)^ |  |  |  |  | **🗸** (D) |  |  | **🗸** |  |  |  |
| 3 ^(40)^ |  |  |  |  | **🗸**(D) |  |  | **🗸** | **🗸** |  |  |
| 4 ^(41)^ | **🗸** | **🗸** |  |  |  |  |  |  |  |  |  |
| 5 ^(42)^ | **🗸** | **🗸** |  |  | **🗸** |  |  |  |  | **🗸** |  |
| 6 ^(43)^ |  |  |  |  |  |  |  |  |  | **🗸** |  |
| 7 ^(44)^ |  | **🗸** |  |  |  |  |  |  |  | **🗸** |  |
| 8 ^(45)^ |  | **🗸** | **🗸** |  |  |  |  |  |  |  |  |
| 9 ^(46)^ |  |  |  |  |  |  |  |  |  |  |  |
| 10 ^(47)^ |  |  | **🗸** |  |  |  |  |  |  | **🗸** |  |
| 11 ^(48)^ |  |  | **🗸** | **🗸** |  |  |  |  |  | **🗸** |  |
| 12 ^(49)^ |  |  | **🗸** |  | **🗸** |  |  |  |  |  |  |
| 13 ^(50)^ |  |  |  |  |  |  |  | **🗸** | **🗸** |  |  |
| 14 ^(51)^ |  |  | **🗸** | **🗸** |  |  |  |  |  | **🗸** |  |
| 15 ^(52)^ |  |  |  |  | **🗸**(D) | **🗸** |  |  | **🗸** |  |  |
| 16 ^(53)^ |  |  |  | **🗸** |  |  |  | **🗸** |  |  |  |
| 17 ^(54)^ |  |  | **🗸** |  |  |  |  |  |  | **🗸** |  |
| 18 ^(55)^ |  |  |  |  | **🗸** | **🗸** |  |  |  | **🗸** |  |
| 19 ^(56)^ |  |  |  | **🗸** | **🗸** |  | **🗸** |  |  |  |  |
| 20 ^(57)^ |  |  | **🗸** |  | **🗸** |  | **🗸** |  |  | **🗸** |  |
| 21 ^(58)^ |  |  | **🗸** |  | **🗸** |  |  | **🗸** |  |  |  |
| 22 ^(59)^ |  |  | **🗸** |  | **🗸** |  |  | **🗸** |  |  |  |
| 23 ^(60)^ |  | **🗸** | **🗸** | **🗸** |  |  | **🗸** |  |  |  |  |
| 24 ^(61)^ |  |  |  | **🗸** | **🗸**(D) |  |  |  |  |  |  |
| 25 ^(62)^ |  | **🗸** |  |  |  | **🗸** |  |  |  |  |  |
| 26 ^(63)^ |  |  | **🗸** |  | **🗸** |  |  | **🗸** |  |  |  |
| 27 ^(64)^ | **🗸** |  |  |  | **🗸** |  |  |  |  |  |  |
| 28 ^(65)^ |  |  | **🗸** |  |  |  |  |  |  |  |  |
| 29 ^(66)^ |  |  | **🗸** |  |  |  |  | **🗸** |  |  |  |
| 30 ^(67)^ |  |  | **🗸** |  |  |  |  |  |  | **🗸** |  |
| 31 ^(68)^ |  |  |  |  |  |  |  |  |  | **🗸** |  |
| 32 ^(69)^ | **🗸** |  | **🗸** |  |  |  |  |  |  |  |  |
| 33 ^(70)^ |  |  |  | **🗸** |  |  |  | **🗸** |  |  |  |
| 34 ^(71)^ |  |  |  | **🗸** | **🗸** |  |  |  |  | **🗸** |  |
| 35 ^(72)^ | **🗸** |  | **🗸** |  |  |  |  |  |  |  |  |
| 36 ^(73)^ |  | **🗸** |  | **🗸** | **🗸** |  |  |  |  | **🗸** |  |
| 37 ^(74)^ |  |  | **🗸** |  | **🗸** | **🗸** |  |  |  |  |  |
| 38 ^(75)^ |  |  |  |  | **🗸**(D) | **🗸** | **🗸** |  |  |  |  |
| 39 ^(76)^ | **🗸** |  | **🗸** |  | **🗸** |  | **🗸** |  | **🗸** |  |  |
| 40 ^(77)^ |  |  |  |  |  |  |  |  |  |  |  |
| 41 ^(78)^ |  |  |  |  |  | **🗸** | **🗸** |  | **🗸** |  |  |
| 42 ^(79)^ |  |  |  |  |  |  |  |  |  | **🗸** |  |
| 43 ^(80)^ |  |  |  |  |  |  |  |  |  |  |  |
| 44 ^(81)^ |  |  |  |  |  |  |  | **🗸** |  |  |  |
| 45 ^(82)^ |  |  |  | **🗸** |  |  |  |  |  |  |  |
| TOTAL | **6** | **7** | **18** | **11** | **18** | **6** | **6** | **10** | **5** | **14** |  |

RAG = Red (low), Amber (moderate), Green (high) rating of data sources (documents) for relevance to the research question and usefulness in terms of their contribution towards determining the IPT

Documents were rated ‘low’ (n=3) if they did not contribute any relevant CMOCs, ‘moderate’ (n=7) if they contributed to 1 CMOC and ‘high’ (n=35) if they contributed to 2 or more CMOCs
