# Supplementary material for: A Realist Scoping Review of Community Nutrition Interventions in the UK: Implications for the ‘Nutrition Skills for Life’ Programme
Source: J Hum Nutr Diet. 2025 Jan 8;38(1):e70008. doi: 10.1111/jhn.70008 (PMC11707723; doi:10.1111/jhn.70008)
Supplement: Supplementary file 4 — Descriptive characteristics of included documents. [file JHN-38-0-s007.docx]

| **Reference, year, where in UK, name of intervention (if specified)** | | **Aim of study, aim of intervention (if specified)**  **Focus on impact of socioeconomic status YES/NO** | **Type of study** | **Target group, sample size (n)** | **Setting/provider (if specified)** | **Theoretical basis, (e.g. substantive theory (ST), behaviour change techniques/model (BCT)) described** | **Main outcomes/ findings** | **Contribution to IPT** |
| --- | --- | --- | --- | --- | --- | --- | --- | --- |
|  | Saeed *et al.,* 2020,  England^(36)^ | Identify psychosocial barriers & facilitators to attending social eating opportunities and ways to make them more attractive, accessible and acceptable  NO | Qualitative  Exploratory  Multiple methods   - Focus groups - semi structured interviews | Older adults >60 years  n=42  Socioeconomic position (SEP) not specified | Older adults living in the community or sheltered housing | None described | Lunch clubs needed to be ‘more than the meal’, opportunity to take part in activities and meet new people, avoid labels of unrelatable social categories, encourage people to take first step into group with another person, provide personal invitation and address self-consciousness when eating. Further work needed to test in real life settings and with groups of different socioeconomic status. | Moderate |
| 2. | Funnell *et al.,* 2018,  England^(37)^ | Investigate expectations, knowledge, behaviour, information sources and gap between expectations and level of satisfaction on advice received about nutrition and vitamin supplementation  NO | Quantitative  Cross sectional survey | Pregnant women  n=133  SEP not specific focus | Routine ante natal appointment | BCT/model- Motivational Interviewing, | Women did not receive timely/accurate advice to enable them to take recommended pregnancy supplements at the optimal time, had misconception that they understood correct use of pregnancy supplements.  Digital intervention prenatally could provide this information and improve supplement uptake | High |
| 3. | Abayomi *et al.,* 2020,  multiple UK sites^(38)^ | Investigate experiences of healthy  eating/ weight management advice during pregnancy  NO | Qualitative  Exploratory  Group discussion and 1 interview | Pregnant or 12 months post-natal  n=32  SEP not specific focus | Users of ante natal care | ST- Theory of planned behaviour  BCT/model- individualised goal setting | Pregnant women want positive messages about what they can/should do rather than not do.  Midwives need to consider their communication with women to maintain their unique relationship when discussing weight.  Ensure consistent message whilst personalising healthy eating and weight management advice.  Midwives need to feel knowledgeable and confident. Digital intervention could improve access to information and empower midwives to provide person centred support. | High |
| 4. | Spyreli *et al.,* 2021,  NI or IOI^(39)^ | Explore how nationwide lockdown due to COVID-19 influenced the food-related decisions of socioeconomically deprived families and understand nutritional challenges  YES | Qualitative  Exploratory  Multiple methods   - Online semi structured interviews - Photovoice, map local area | Parents of 2-17-year olds  n=12  Economically disadvantaged | Low SES, Families on low income | None described | Dietary changes observed during ‘lockdown’- more home-made meals, increase in unhealthy snacking, meal planning due to fewer shopping trips. Infrequent takeaways helped with finances School meals payment scheme and food donations lessened expenditure on food and contributed to food security for low income families. | High |
| 5. | Lovelace *et al.,* 2015,  England^(40)^ | Explore food choices of low-income families with pre-school children; to understand socio-economic and environmental influences and constraints  YES | Qualitative  Exploratory  Semi structured interviews | Parents of preschool aged  n=11  Economically disadvantaged | Families did not own home, received income support and Healthy Start | None described | Mothers access information on introducing solid foods but mainly introduce them earlier than recommended. Confusion exits regarding benefits of, and how to access free vitamins. Brand loyalty led to belief that manufactured baby foods must be ‘good’ for toddlers. Complexity of health promotion messages need to be translated into useful menus for parents by HCPs and others working with families. Parents’ diets may improve as a result of them improving their child’s diet. | High |
| 6. | Gatley *et al.,* 2014,  UK combined with a non-UK site^(41)^ | Compare influences on attitudes and behaviours relating to domestic cooking habits in France and Britain and how (including skills employed) food items are transformed into culturally acceptable meals.  NO | Qualitative  Exploratory  Semi structured interviews | Adults  n=27  SEP not specific focus | Adults living in England (n=13) or France (n=14) | None described | People lack time to cook and increasingly relied on a mix of both raw and convenience-type foods. Cooking habits reflect wider economic, social and cultural changes. Less time cooking in the home may negatively impact on intergenerational transfer of cooking skills and undermine confidence to cook. Less pride in deep rooted national cuisine and its continuation in British respondents than French counterparts. | Moderate |
| 7. | Lavelle *et al.,* 2016,  NI or IOI^(42)^ | Explore how individuals define cooking from ‘scratch’, and their barriers and facilitators to cooking with basic ingredients.  NO | Qualitative  Exploratory  Semi structured interviews | Adults  n=27  SEP not specific focus  Sample were highly educated | Adults male (n=10) and female (n=17) | None described | Interventions should focus on practical session to increase cooking self-efficacy; highlight the importance of planning head and teach methods such as batch cooking and freezing to facilitate cooking from scratch. Opportunities to experiment, fail and learn from failure can increase skills and boost cooking self-efficacy. | High |
| 8. | McFadden *et al.,* 2014,  England. ***Healthy Start***^(43)^ | Evaluate ‘Healthy Start’ to determine whether food vouchers can contribute to reducing nutritional inequalities. ‘Healthy Start’, a UK targeted food subsidy programme which provides credit for fruit, vegetables, milk and vitamins, aims to provide a nutritional safety net and improve nutrition for low income families.  YES | Qualitative  Evaluation  Multiple methods   - Focus groups - Online consultation - Participatory workshops | Pregnant or parents of child under 4 years  n=729 and Health care practitioners n=49  Economically disadvantaged | Families in receipt of Healthy Start  Health and Social Care Practitioners | None described | Food subsidy schemes provide an important nutritional safety net and potentially improve nutrition for pregnant women and young children living on a low income. The scheme increased quantity and range of fruit and vegetables used, improved diet quality and established good habits for the future. Erosion of subsidy value relative to the rising cost of food, lack of access to registered retailers and barriers to registering could compromise impact. | High |
| 9. | Clifford Astbury *et al*., 2019,  multiple UK sites^(44)^ | Determine whether consumption of home-prepared food is necessary for high dietary.  NO | Quantitative  Cross sectional analysis of UK dietary survey data  Food and drink diary | Households randomly selected and a minimum of one adult and one child took part.  n=1063  SEP not specified | Adults previously recruited to NDNS  Study sample more affluent than general NDNS sample | None described | A healthy diet could be achieved with little contribution from home-prepared foods and therefore hoe prepared foods should not be presented as a prerequisite to high quality diet. However, the sample were more affluent than the general survey sample and the low home preparation group consumed more sugar and salt than the high home preparation group. | Low |
| 10. | Pallan *et al.,* 2012,  England^(45)^ | Access key contextual data to inform the development of an obesity prevention programme targeting South Asian children  NO | Qualitative  Exploratory  Focus groups | UK South Asian population  Stakeholders including parents, school staff, community leaders  n=68  SEP not specified | Schools participating in obesity prevention programme | ST- Ecological systems theory/model | Understanding differing cultural contexts is crucial  to successful childhood obesity intervention. Otherwise,  opportunities for intervention impact will be missed. For South Asian communities, understanding the central role of religious practices is essential. Other themes were child preference, sedentary activities, parental role  models, constrained parental time, unhealthy school food, access to leisure facilities, fast food availability, food marketing and safety. | High |
| 11. | Van Kesteren *et al.,* 2020,  England^(46)^ | Analyse and understand the cooking practices of mothers living in areas with different levels of deprivation.  YES | Mixed method  Comparative case study  Practice oriented interviews  Ethnographic observations  Survey  Food and drink diary | Mothers of young children  n=310 surveyed  n=25 interviews  n=6 ethnographic observations  Different socioeconomic contexts explored | Community based  Mothers as tend to be at the centre of what household eats | ST- Practice theoretical/ orientated approach | Beyond rational choice/intention, cooking is best understood as a practice produced by a web of unthinking elements that align with socioeconomic differences. Increasing education and awareness without engaging with context e.g. social inequities, cannot be adequate to increase’ healthy eating’ and could make inequalities worse. Interventions must consider complexities & avoid a narrow focus on individual choice or ‘victim blaming’ those who face the greatest challenges. | High |
| 12. | Ohly *et al*., 2012,  England^(47)^ | Explore factors influencing parental food choices and assess their requirements for healthy eating support prior to the development of a tailored intervention.  NO | Quantitative  Cross sectional analysis Questionnaire | Parents of children 2-5 years  n=261 | Childrens centres in rural and urban locations | None described | Most important factors that influenced food choice of parents were healthiness, taste, freshness and quality. The most popular ideas for intervention were recipe ideas, practical ways to encourage children to eat well and overcoming fussy eating. The last popular option was home based support for parents. Less well-educated parents wanted to learn more about what a healthy diet meant, how to understand food labels, budge for food and portion sizes. There was demand for healthy eating support & children’s centres are an ideal setting for this. | High |
| 13. | Warren *et al.,* 2017,  Wales. ***Eat Well Keep Active***^(48)^ | To evaluate the feasibility, acceptability and perceived efficacy of the novel ‘Eat Well Keep Active’ intervention programme.  ‘Eat Well Keep Active’, a brief midwife led intervention, aims to facilitate healthful dietary and physical activity behaviours.  NO | Qualitative  Evaluation  Semi structured interviews | Pregnancy of less than 16 weeks duration. First baby  n=20  SEP not specific focus  All women were in employment | Midwifery led care | ST- Self Determination Theory  BCT/model- Motivational Interviewing, individualised goal setting | Women during pregnancy welcomed individualised discussions regarding diet and exercise and reported it positively influenced their health behaviours. The intervention was designed to be incorporated into existing antenatal provision and therefore financially feasible. Women reported a sense of ownership of goal set and levels of confidence in their ability to change their behaviour improved, Support from significant others was also important and goals were valued by partners. | High |
| 14. | Neale *et al*., 2012,  England^(49)^ | Provide new insights into heroin users’ eating patterns in order to inform nutritional interventions.  NO | Qualitative  Exploratory  Semi structured interviews | Current or ex heroin users  n=40  Varied socioeconomic backgrounds | Community and residential drug services, pharmacies or peer support groups | None described | Inadequate and infrequent food intake, low consumption of fruit and vegetables, elevated consumption of sugar and eating disorders were evident. Nutrition was better for those in residential settings and in recovery when pleasure and interest in eating returned. After reducing drug use, managing to cook and eat a balanced diet can have important therapeutic value by improving  health, self-esteem and social relationships, generating  pleasure and offering financial savings. | High |
| 15. | Papadaki *et al.,* 2016,  England^(50)^ | Explore adult employees' perceptions of ability and barriers to follow the Med-Diet; preferences for setting and reviewing goals and receiving feedback on achievement during an intervention promoting the Med- Diet, and expectations of an Internet based, workplace MedDiet intervention.  NO | Qualitative  Exploratory  Focus groups | Adults  Workplace setting  n=29 | Workplace settings  Involving family support | BCT/model- individualised goal setting | Workplace interventions to promote the Mediterranean Diet (MD) should utilise a tailored, participant led approach to setting MD goals and reviewing and receiving support on goal attainment and develop social support mechanisms. Overall there was preference for reviewing goals by using a combination of smartphone app or website and assistance from a nutrition expert and receiving feedback from a HCP via app, website, text or at a face t0 face session. Workplace activities should eb combined with a family orientated intervention. | High |
| 16. | Wood, 2017,  England^(51)^ | Highlight issues that may affect older people and lead to under-nutrition, and provides simple preventative measures.  NO | Narrative piece | Older adults  n/a | Community nursing and domiciliary care | None described | Lack of good local shops and affordable healthy products, poor mobility and underlying health conditions, in addition to social isolation and limited resources can make good nutrition an impossible task for older people. Social eating through lunch clubs can address loneliness, isolation and nutrition. | High |
| 17. | Bispo *et al.,* 2013,  England^(52)^ | Characterise the nutrition and health of Brazilians living in Bournemouth, England, and changes in lifestyle after migration.  NO | Quantitative  Cross sectional analysis  Structured questionnaire  24-hour dietary recall  Food frequency questionnaire | Adult migrants from Brazil to the UK  n=111  SEP not specific focus | Community | None described | Health and nutritional benefits in immigration erode over time. Weight gain was reported post migration, with increased consumption of food rich in calories and low in nutrients. Obesity and risk of metabolic complications increased with time living in England. | High |
| 18. | Garcia *et al.,* 2017,  Scotland. ***Eat Better Feel Better***^(53)^ | Evaluate the effectiveness of ‘Eat Better Feel Better’ community-based cooking skills programme in increasing confidence in cooking and tackling the barriers of time, cost, waste, and knowledge of cooking and healthy eating in the short (post-intervention) and mid-term (3–4 months follow-up).  NO | Mixed method  Evaluation  Pre, post and follow up questionnaire  Qualitative questions included in post intervention questionnaire | Adults  n=62 pre and post,  n=17 also completed 3-4 month follow up | Community | ST- Social Cognitive Theory | Programme had positive effects on participants’ cooking skills confidence, helped manage time, and reduce barriers of cost, waste and knowledge. Resulted in increased knowledge of food labels, portion sizes and importance of a balanced diet. Attending more sessions was important for increasing confidence. Educational aspects and learning increased enjoyment and value. Socialising increased enjoyment. Some claimed improved cost/budget and time saving through the use of leftovers to make new meals, decreasing food waste and planning/preparing more meals. Bought less processed food and more fruit and vegetables, less impulse buying. | High |
| 19. | Campbell- Jack *et al.,* 2020,  England. ***Holiday Activities and Food (HAF)***^(54)^ | Evaluate the ‘Holiday Activities and Food’ (HAF) Programme.  HAF aims to provide free holiday club places for children and young people aged 4-16. HAF provides healthy food, structured physical activity and opportunities for children and parents/carers to develop nutrition knowledge and food skills.  NO | Mixed method  Evaluation  Contribution analysis approach  Survey  Telephone interviews  Case study observations | Co-ordinator interviews (n=21), surveys for provider (n=527), children (n=1131) and parents (n=370) | Schools, voluntary organisations and private organisations | BCT/model -Logic Model described | Nutrition education (NE) worked when it was part of existing activities and where experiential learning approach applied. ‘Fun activities’ enabled YP to socialise and increased their confidence. Cost of food, sufficient trained staff and food to cover fluctuating attendance, meeting dietary requirements and sources ingredients were concerns. Parent involvement was important to parents and children. NE could be further facilitated e.g. linking parents with food aid, community hubs. Running in nearby, familiar places helped attendance. Staff were surprised when children ate e.g., fruit and veg. Onsite cooking embedded NE messages. Suppliers did not always deliver food that met standards e.g., ‘coke and Haribos’, Foodbank providing trays of doughnuts. Barriers to delivery were lack of cooking facilities, lack of training for staff and NE being informal rather than formal sessions. | High |
| 20. | Hutchinson *et al.,* 2016  England. ***Ministry of Food***^(55)^ | Evaluate the impact of the Jamie Oliver ‘Ministry of Food’ (MoF) cooking course in relation to changes in F&V and snacks consumed and confidence in cooking.  MoF aims to improve confidence to cook and increase consumption of homemade food.  NO | Mixed method  Evaluation  Pre, post and follow up questionnaire  Structured telephone interviews | Adults  n=462 completed pre, post and 6 month follow up questionnaires  24% came from deprived areas | Community venues | None described | Participants from deprived areas valued learning new recipes & ways to cook from basic ingredients. Learnt knife skills, preparing vegetables, seasoning food and food hygiene. Claimed improved knowledge on healthy eating, benefits of increasing F&V and reducing saturated fat intakes. Gained nutrition knowledge, value of health food, healthier ways to cook and food label reading. Helped with portion control, cooking batch portions and reducing waste. Decreased social isolation as ‘got them out of the flat’. Enjoyed working as a group and meeting new people. Staff interaction and group atmosphere enhanced learning and confidence for those with low literacy levels. Convenient location & store tour enabled awareness & access to affordable, healthy ingredients. | High |
| 21. | Stevens *et al.,* 2021,  England^(56)^ | Explore dietary habits, lifestyle patterns and barriers to healthy eating and the associations between nutrition knowledge (NK), sleep quality and weight change during the post-partum period.  NO | Quantitative  Cross sectional survey | Post-partum mothers with baby under 1 year  n=228 survey responses  n=34 completed optional dietary recall  Sample were highly educated | Community  Children’s centres | None described | Limited nutrition information provided for women in post-partum period (PPP) as focus is on baby, not new mother. Fatigue, lack of time and stress were barriers to healthy eating (HE) & greater in women from more disadvantaged backgrounds. Gaining an understanding of the level of nutrition knowledge (NK) & recognising that ‘mistaken or false beliefs’ may exist will be important in the development of effective nutrition strategies. HE advice needs to be practical and realistic, taking into consideration the limited time available to dedicate to HE among competing priorities. Further research is needed to explore the role of NK on PP weight retention and eating behaviours. | High |
| 22. | Avgerinou *et al.,* 2019,  England^(57)^ | Explore views & dietary practices of people at risk of malnutrition and their carers; identify gaps in knowledge, barriers and facilitators to healthy eating in later life; & explore potential interventions for malnutrition in primary care.  NO | Qualitative  Exploratory  Multiple methods   - Semi structure interviews - Focus groups | Older adults >75 years of age and at risk of malnutrition n=24, and informal carers n=9. | Community dwelling | None described | Older people at risk of malnutrition rarely recognise appetite or weight loss as a problem, perceiving being thin is healthy and ‘snacking’ is unhealthy. Changes in household composition, physical or mental health conditions and cognitive impairment can lead to inadequate food intake. Lack of specialist training in nutrition and gap in HCP knowledge limits the dietary advice given to older adults. Education is needed to inform older people of their dietary requirements and tailor support to individual needs. Carers acknowledge malnutrition as a problem but lacked knowledge of how best to respond to weight loss therefore, interventions that aim to support nutrition in frail older people should also provide education to caregivers. | High |
| 23. | Ord and Monks, 2022,  England^(58)^ | Explore how food poverty impacted on youth work, youth workers’ understanding and awareness of food poverty and whether and how they were responding to the issues.  YES | Qualitative  Exploratory  Semi structured interviews | Youth workers.  n= not specified  Young people in or at risk of food poverty | Youth centres | None described | Youth workers are part of a ‘community response’ that addresses food poverty & builds social capital through social relationships associated with food by 'eating together'. This can combat stigma and abjection through critical consciousness and political education. Youth centres (YC) need to be less isolated from other services, food provision could be enhanced if co-ordinated with other community food provision/ networks. Shared meals offered opportunity to build togetherness and community spirit ‘looking after each other’. Youth workers felt a strong moral obligation to feed young people (YP) when hungry & recognised structural foundations of the problem which they could raise awareness of amongst YP. Food poverty not only affects the quantity /quality of food YP can access but relationships are also maintained and sustained through the provision of food and sharing of meals. If tis is denied, this can bring about stress, is demoralising and can directly impact mental health and wellbeing, feeling stigmatised and having low self-worth. Provision of food in YC is an opportunity for important conversation about nutritional value: social, emotional and psychological aspects and structural and political dimensions of food. | High |
| 24. | Mills *et al.,* 2017,  England^(59)^ | Assess whether frequency of consuming home cooked meals was cross-sectionally associated with diet quality and cardio-metabolic health.  NO | Quantitative  Cohort study  Plasma markers  Food frequency questionnaire | Adults  n=12,434  SEP not a specific focus | Community dwelling | None described | Eating home cooked meals more frequently was associated with greater adherence to DASH and Mediterranean diets, greater fruit and vegetable intakes and higher plasma vitamin C, in adjusted models. Those eating home cooked meals more than five times, compared with less than three times per week, consumed more fruit and more vegetables daily. More frequent consumption of home cooked meals was associated with greater likelihood of having normal range BMI and normal percentage body fat. Associations with HbA1c, cholesterol and hypertension were not significant in adjusted models. Those consuming home cooked meals more than five times, compared with less than three times per week, were less likely to have overweight BMI and less likely to have excess percentage body fat. | High |
| 25. | Relton *et al.,* 2020,  England. ***Fresh Street***^(60)^ | Develop and test the feasibility and acceptability of ‘Fresh Street’, a voucher scheme that targeted areas rather than individual families.  ‘Fresh Street’ aimed to: increase fresh fruit and vegetable consumption; encourage new purchasing, food preparation and eating patterns; reduce food poverty and improve health outcomes.  YES | Qualitative  Evaluation  Rapid ethnographic assessment  Informal conversations | Adults (n=141) and children (n=63) from 97 households  SEP a focus and prevention of food poverty | Households | None described | Householders reporting eating fruit once a day or more increased over the 10-month duration. Some households reported cooking from scratch more. Many mentioned using, sharing and saving the recipes provided with the vouchers. Households who joined the scheme reported that the vouchers made them think about what they were eating, and prompted them to buy and eat more F&V and to not waste the vouchers. Many reported new purchasing patterns. | High |
| 26. | Lucas *et al.,* 2020,  multiple UK sites^(61)^ | Explore the experiences of healthcare professionals in supporting young women around eating and moving during and after pregnancy.  NO | Qualitative  Exploratory  Interviews (telephone or face to face) | HCPs supporting women in pregnancy and post-natal period  n=17 healthcare professionals | Staff working women during and after pregnancy | BCT/model -COM -B model | For HCPs, providing support around eating was led by tacit knowledge. Without specific knowledge of how to address eating with young women, HCPs might avoid discussing eating habits. HCPs trained in behaviour change techniques were confident to communicate with young women around eating and moving. Those who had not received training, based conversations on 'common sense' or experience gathered over time and sometimes directly challenge young women. Others expressed some anxiety about discussing weight with young women- not feeling well equipped and coming over as judgemental. For some HCPs, their immediate focus was more on infant health, rather than young women's eating habits. Relationships of trust with young women needed to be established before work on health behaviours could begin. If HCPs were supported with knowledge and communication skills and had time, they were more motivated to discuss health behaviours. The pressure of time and cuts on healthcare professionals’ roles (physical opportunity) may stifle their capability to provide the optimum support for young women. | High |
| 27. | Bagwell, 2014,  England. ***Healthier Catering Commitment***^(62)^ | Explore issues arising from an evaluation of the ‘Healthier Catering Commitment’ (HCC).  HCC recognises businesses in London that demonstrate commitment to reducing levels of saturated fat, salt and sugar in foods sold on their premises.  NO | Mixed method  Evaluation  Survey  Interviews (telephone or face to face)  Focus groups | 77 businesses implementing the scheme  Interviews with businesses (n=10) and customers (n=28)  SEP not a specific focus | Independent food businesses | None described | Interventions, which sought to change consumption behaviour by the provision of information on healthier food and by offering healthier alternatives, resulted in the provision of healthier food for some. These tended to be the more affluent, and often already more health-conscious customers however, who were able and willing to pay the extra costs involved. The healthier alternatives were positively resisted in some of the more deprived communities, particularly where they resulted in increased prices. Thus, it seems that the healthier alternatives promoted by healthier catering schemes may well be improving the health of the already more health-conscious middle classes, but are unlikely to be having much of an impact on the more disadvantaged members of our society. By improving the health of those that can afford to take advantage of these healthier choices, they are possibly unwittingly widening the gap in health inequalities. | High |
| 28. | Jarman *et al.,* 2015,  England^(63)^ | Explore mothers’ use of overt and covert control practices in early childhood, the role of food neophobia in predicting changes in control practices, and associations between these factors and with the children’s quality of diet.  NO | Mixed method  Exploratory  Survey  Telephone questionnaire  Food frequency questions  Focus groups | Mothers surveyed (n=228) and interviewed (n=29) | Parents of children 0-7 years | None described | Mothers who increased their use of overt control had children whose level of food neophobia also increased. Mothers who used more covert control had children with better quality diets, and mothers who increased their use of covert control over the two year follow-up had children whose diet quality improved. Mothers suggested that feeding young children was stressful and that control was often relinquished in order to reduce conflict at mealtimes. Children whose mothers used less covert control consumed crisps, cakes and biscuits, and soft drinks more often, and drank water less often, than children whose mothers used covert control more often. Interventions should be supportive and tailored towards empowering mothers to feel able to use more covert than overt control as a way of encouraging their children to have healthier diets. | Moderate |
| 29. | Heslehurst *et al.,* 2014,  Systematic review^(64)^ | Systematic review to identify the determinants of healthcare professionals’ behaviours in relation to maternal obesity and weight management.  NO | Review 25 studies  (14 qualitative, 11 quantitative and 1 mixed methods) | HCPs caring for women during pregnancy | Pregnancy  HCPs | ST- Theoretical Domains Framework  BCT/model - Motivational Interviewing, individualised goal setting | HCPs were motivated to address weight during pregnancy but generally lacked formal training. Good communication skills were deemed important, yet they lacked confidence and felt uncomfortable initiating discussions about weight. The belief that they lacked sensitive communication skills included uncertainty about the appropriate language and terminology to use. Practical resources were also desired to facilitate communication. Discussing women’s weight status was consistently described as being sensitive or emotive. HCPs believed that these discussions would result in negative responses including offence, upset, embarrassment, stigma, victimization, fear, blame, judgement or defensiveness. Some also believed these discussions would negatively impact on the relationships they had with women, result in complaints, and women would disengage from care at that maternity service. These beliefs resulted in negative emotions for healthcare professionals, such as anxiety, embarrassment, worry and upset. Generally, they felt they lacked the knowledge, behaviour change skills and confidence to provide weight management support. | High |
| 30. | Lavelle *et al.,* 2017,  NI or IOI^(65)^ | Examine the role of enjoyment in cooking, perceived confidence to cook a recipe, and perceived difficulty of meal preparation on the intention to cook from basic ingredients.  NO | Quantitative  RCT  Adapted cooking skills questionnaire | Adults  n=141  SEP not a specific focus | Adults  Community dwelling | ST- Self Determination Theory, Self-efficacy Theory  BCT/model - individualised goal setting | There was a significant increase in enjoyment, confidence and intention to cook from basics again and a decrease in perceived difficulty after the experiment in all conditions. .  Cooking interventions should focus on practical cooking and increasing participants' enjoyment and confidence during cooking to increase intention to cook from basic ingredients at home. As there were no differences between the conditions on the various measures, by allowing participants choose their method, this would increase autonomy, a key aspect of adult learning. | High |
| 31. | Hardcastle and Blake, 2016,  England^(66)^ | Understand the influences and perceived barriers underlying food choices and eating patterns in mothers in an economically disadvantaged community; examine the impact of an after-school intervention on food choices, confidence and cooking skills.  YES | Qualitative  Exploratory  Semi structured interviews | Mothers of school aged children  n= 16 first interviews and n=4 second interviews  Economically disadvantaged populations | After school club | ST- Ecological systems theory/model, Social Cognitive Theory | There was a positive influence of the after-school cooking intervention on children and their families in cooking skills, promoting healthier cooking methods and increasing confidence to prepare homemade meals. The intervention was perceived to improve nutritional knowledge, cooking skills and increasing confidence to make healthy and tasty homemade meals. Participants' were surprised that healthy meals could be made quickly from scratch and could be tasty pointing to the importance of an active and experiential component in healthy eating interventions. Most participants displayed adequate knowledge of HE but were locked into familiar eating practices that were not necessarily healthy but lacked the skills or confidence to make different meals. Hands-on cookery interventions may be capable of changing ingrained eating practises and food choices. | Moderate |
| 32. | Poland *et al*., 2013,  England^(67)^ | Assess the potential for creating a “health cafe´” to facilitate healthier lifestyles.  YES | Mixed method  Exploratory  Documentary analysis  Focus group  Survey  Semi structured interviews | Adults  n=121 adults surveyed  n=18 focus group participants  n=13 stakeholder interviews  SEP not specified | Community | None described | Respondents’ views on a healthy diet focused mainly around eating five-a-day, low fat foods & a balanced diet. Not knowing where to go for healthy food, HE was too expensive, and family and friends would not go to healthy eateries with them were barriers. Accessibility and comfort of a ‘health café’ were more important than healthy eating. It had to be inviting and accessible to all. Using the word “health” in the label was off-putting and “healthy food” was equated with costliness. Most stakeholders felt the facility should enable people to feel empowered to make healthy choices as community partners rather than have health messages “imposed” on them. All agreed food provided should be healthy but the facility should be an inclusive, multi-purpose hub that widened access to local life-enhancing opportunities. The public and stakeholders would need to be confident and trust that the facility had a long-term future if they were to engage. | High |
| 33. | Philpin *et al*., 2014,  Wales^(68)^ | Investigate factors influencing nutritional care provided to residents in two different types of residential care settings and the experiences of residents and their informal carers.  NO | Qualitative  Exploratory  Multiple methods  -Focus groups  -Semi structured interviews  -Observations and field notes | Care home staff  n= 15 catering staff at focus groups  n=4 managers/assistant manager, n=16 residents and n=10 informal carer interviews  SEP not specified | Older adult care settings | None described | The shared meaning of mealtimes for residents, informal carers and staff was constructed from each group’s sociocultural background, family experiences and memories, and was integral to residents’ sense of normality, community and identity. The geographic and spatial context of care homes influenced residents’ mealtime experiences, through conversation and companionship, reciprocity and mutual support in the social relationships between residents as they helped each other by passing condiments, pouring drinks for each other and ensuring that everyone had what they needed. There were complexities around official views of a ‘healthy’ diet & important aspects of food, such as pleasure (especially in terms of ‘tastiness’) and links to home and family. Shared understandings of the meanings attached to food/mealtimes were important for community and homeliness in the care homes, & residents’ sense of identity and self. A need for appropriate training for care home staff on nutrition & special dietary needs of older people was identified and addressed. | High |
| 34. | Mills *et al*., 2020,  UK combined with a non-UK site^(69)^ | Explore the concept of ‘home cooking’, combining qualitative data from two previously completed studies to undertake a new secondary analysis using the Framework Method.  NO | Qualitative  Exploratory- secondary analysis  Multiple methods  -Focus group  -Semi structured interviews | Adults  n=53 focus group participants  n=18 semi structured interviews  SEP a key focus using | Adults  Community dwelling | None described | ‘Home cooking’ was defined as preparing a meal from scratch, cooking with love and care, and nostalgia. Benefits of ‘home cooking’ were generally social, cultural, and emotional gains, and dietary advantages from using raw foods or traditional cooking techniques that were not reliant on highly processed ingredients. Participants described pleasure in cooking a meal for others, and a sense of satisfaction in creating a dish from basic ingredients. ‘Home cooking’ was important for fostering strong and loving personal connections. Participants did not specifically link diet quality or physical health with ‘home cooking’, and instead focused more on social and emotional dimensions, such as nostalgia. Public health nutrition campaigns should aim to integrate the promotion of cooking with broader dietary guidance, such as recommendations for the consumption of wholegrains and fruit and vegetables. Cooking at home may offer diet, health and social benefits, & public health initiatives should continue to promote cooking at home. | High |
| 35. | Lambie-Mumford, 2012,  England. ***New Deal for Communities***^(70)^ | Provide insights from research that explored how the ‘New Deal for Communities’ (NDC) partnerships reacted to issues of food poverty and what, if anything, they did to try to alleviate them.  YES | Qualitative  Evaluation  Multiple methods  -Semi structured interviews  -Focus group  -Documentary analysis | Adults  n=9  Low SEP  Regeneration areas | Adults  Community dwelling | None described | Food poverty was not a strategic priority for New Deal for Communities (NDC) partnerships. While some activity did occur on food issues, outcomes were limited and barriers to food access remained. Work to pursue better retail in the areas was not pursued by the NCD despite strategic priorities being improved nutrition through better access to food and a recognition of poor local shopping provision and the need to develop opportunities for food shopping, growing and cooking. An approach to food poverty framed in terms of HE runs the risk of promoting an idea of food as primarily a means to better health and physical efficiency, and can detract from a broader appreciation of how constrained food experiences limit social participation. | High |
| 36. | Mills *et al.,* 2018,  England^(71)^ | Identify sociodemographic characteristics associated with frequency of consuming home cooked meals and meals from different out of home sources, namely takeaways, pre-prepared ready meals and eating out.  NO | Quantitative  Secondary analysis of large cohort study data  questionnaire and clinical measurements | Adults  n=11326  SEP not main focus | Adults  Community dwelling | None described | Eating home cooked meals more frequently was associated with being female, older, of higher Socio-Economic Status (SES) (measured by greater educational attainment and household income) and not working overtime. Consuming more takeaways was associated with lower SES, & eating out more frequently was associated with higher SES and working overtime. The majority ate home cooked meals as their main meal at home more than twice per week, whereas few ate ready meals or takeaways more than twice per week. Higher SES was associated with a higher frequency of eating home cooked meals and meals out, and a lower frequency of consuming takeaways. Health promotion messages regarding the potential negative implications of takeaways for diet and health may have been differentially adopted according to SES, which could lead to widening of diet-related health inequalities. | High |
| 37. | Moore *et al.,* 2012,  multiple UK sites^(72)^ | Explore knowledge of the UK weaning guidelines and the sources of weaning advice used and found most influential by UK first-time mothers, and associations between sources of advice, knowledge of the guidelines and weaning timing.  NO | Mixed method  Exploratory  Survey  Free text responses keyword and thematic content analysis | Parents of children in early years  n=1348  Sample were highly educated | Parents of children 0-7 years | None described | First time mothers seek weaning information from multiple sources, much of which is conflicting. Younger, less well-educated mothers are influenced by older family members leading to weaning earlier than recommended. HCPs should signpost parents to reliable information online. Further work to investigate challenges with conveying and receiving/interpreting messages, evaluation of weaning interventions and consideration for how to involve education of significant others such as baby’s grandmother are needed. | High |
| 38. | Trewern *et al.,* 2022,  England. ***Sparking Change***^(73)^ | Evaluate the effectiveness of ’Sparking Change’ a multi-component behavioural intervention pilot implemented by a UK food retailer.  ‘Sparking Change’ aimed to support a small target community of customers and retail employees to adopt and maintain more sustainable food behaviours.  NO | Mixed method  Evaluation  Survey  Focus groups | Adults  n=92 pre and post,  n=68 also completed 3 month follow up  n=6-15 attended focus groups  SEP not specified | Adults  Community dwelling | ST- Practice theoretical/ orientated approach  BCT/model COM -B model | Intervention mitigated individual barriers to change, positively impacted on awareness, intention and behaviour which lasted beyond the intervention. Participants reduced meat consumption, food waste and increased cooking from scratch. Online ‘ask the expert’ videos and product samples worked well, recipes and cook-along sessions worked less well. Behavioural interventions offer a positive opportunity to influence behaviour change, however, structural and cultural | High |
| 39. | Coupe *et al*., 2018,  England^(74)^ | Identify how to tailor lifestyle interventions to low socio-economic populations to improve outcomes, from the healthy lifestyle intervention service providers’ and service users’ perspectives.  YES | Qualitative  Exploratory  Multiple methods  -Semi structured interviews  -Group observations | Providers and attendees of healthy lifestyle intervention  n=25  Focus on low socioeconomic populations | Staff who facilitate healthy lifestyle intervention and those who attend. | BCT/model- individualised goal setting | Challenges were; managing diversity- delivering a generic intervention to diverse populations with regards to knowledge, language and literacy, and cultural diversity; and environmental issues such as cost, access and availability of food (and leisure facilities) and ‘life gets in the way’. Interventions should encourage behaviour change techniques such as goal settings, rather than information provision alone, be mindful of cost, cultural diversity, language and literacy barriers and potential for disengaging seldom heard groups. | High |
| 40. | Kelly and Ogden, 2016,  England^(75)^ | Compare looked after and non-looked after young people’s early childhood attachment and explore the role this might play in predicting any eating related problems they may display.  NO | Quantitative  Cross sectional design  Online questionnaires x 5 | Young people aged 16-25 in care  n=64 (32 in care and 32 matched from non-care settings) | Young people (YP) in local authority care settings | ST- Attachment Theory | Looked after YP had poor level of early childhood attachment, higher levels of picky eating and food disgust and a more detached relationship with food. This may lead YP to use eating behaviour both as a defensive mechanism to protect against unreliable food environments and to maintain their sense of wellbeing. HCPs and carers should build reliable, secure relationships with YP to instil a sense of trust which may reduce eating problems in the future. | Low |
| 41. | Bridge *et al.,* 2019,  England. ***Health, Exercise, Nutrition for the Really Young (HENRY)***^(76)^ | Investigate the impact of the HENRY programme upon participating families, identify the potential mechanisms by which the programme achieves positive dietary changes.  HENRY aims to improve diet, exercise, parental skills and emotional health and wellbeing outcomes for children under 5 and their parents.  NO | Qualitative  Evaluation  Multiple methods  -Focus groups  -Telephone semi structured interviews | Mothers of children in their early years  n=39  SEP not specified | Children’s centres | BCT/model - individualised goal setting, Theory of change described | Parents reported enhance self-efficacy and confidence in their ability to encourage healthier behaviours. Mechanisms such as mutual support, a non-judgemental and partnership approach, being listened to by facilitators and group discussion and encouragement to identify their own ideas, focusing on small, manageable steps and a fun, interactive delivery style. | High |
| 42. | McGowan *et al*., 2016,  NI or IOI^(77)^ | Test a model of diet quality by assessing the influence of socio-demographic, nutrition knowledge- and psychological-related variables alongside perceived cooking skills (CS) & food skills (FS) abilities.  YES | Quantitative  Cross sectional survey via computer assisted personal interviewing | Adults aged 20-60  n=1049  SEP not specified |  | ST- Social Cognitive Theory, Theory of planned behaviour | Cooking skills (CS) and food skills may not lead directly to healthier dietary choices due to many other factors at play. However, nutrition knowledge and CS appeared to be linked to lower saturated fat consumption and those identifying as better cooks were more likely to have high fibre intake. Interventions to improve diet quality should focus on cooking identify and health motivations as well as skills. Targeting males, younger adults, those with limited education is advocated. | Moderate |
| 43. | Lara *et al.,* 2015,  England^(78)^ | Assess understanding, acceptability and preference for two graphical displays of the Mediterranean diet (MD); and feasibility of a brief MD intervention and cost of adherence to this diet.  NO | Quantitative  RCT  Food intake diaries  Questionnaires | Older adults  n=55  SEP not specified | Community dwelling | None described | The Mediterranean Diet (MD) was highly acceptable as a model of healthy eating for older adults. An Educational Group Session promoting the MD was acceptable and showed improvement in dietary pattern could be achieved without incurring additional costs. The need to understand effective behaviour change techniques associated with dietary improvement was highlighted. | Low |
| 44. | Willis *et al.,* 2012,  England. ***HENRY***^(79)^ | Report qualitative data collected from staff as part of the evaluation of HENRY, and examine the impact of training upon the Centres and staff teams.  NO | Qualitative  Evaluation  Semi-structured interviews  Comments box | Children’s Centre managers  n=12 interviews  160 comments slips  SEP not specified | Childrens centre staff caring for children 0-7 years | ST- Self-efficacy Theory | Positive and lasting lifestyle effects can be achieved by brief training courses involving Children’s Centre staff teams. Changes to centre policy and practice, provision of age appropriate portion sizes and introduction of healthy snacks, strengthening of team working and increased staff confidence and enhanced skills when working with families around lifestyle change. Training also induced changes within the staff’s personal lives. | Moderate |
| 45. | Thompson *et al.,* 2017,  England^(80)^ | Understand how influences, risk and vulnerability play out in relation to food behaviour, examine their (older bereaved men) everyday food practices and experiences, and explore their attitudes, behaviour and knowledge concerning food and nutrition.  NO | Qualitative  Narrative approach  Unstructured interviews | Older bereaved men, average age 80 years.  n=20 | Community dwelling | None described | Social isolation increased food vulnerability. Without social opportunities to eat, eating became a necessity rather than a pleasure. Shopping and cooking for one presented a challenge. Taking part in social activities where food could be consumed provided men with a sense of mutuality and support, reducing potential for food vulnerability. Integrating financial, transport and social networks as well as cookery classes, may be needed to address complex factors at play. Future studies should explore greater risk of food vulnerability for bereaved men in more deprived communities. | Moderate |
