# Supplementary material for: A Realist Scoping Review of Community Nutrition Interventions in the UK: Implications for the ‘Nutrition Skills for Life’ Programme
Source: J Hum Nutr Diet. 2025 Jan 8;38(1):e70008. doi: 10.1111/jhn.70008 (PMC11707723; doi:10.1111/jhn.70008)
Supplement: Supplementary file 5 — Data for consolidated CMOCs. [file JHN-38-0-s004.docx]

**Supplementary file 5. Data for consolidated CMOCs.**

170 Context-Mechanism-Outcome Configurations (CMOCs) were originally recorded. CMOCs or ‘programme theories’ are building blocks of broader, integrated theory about how complex programmes are thought to work (or not). These CMOCs have also been referred to by others as ‘explanatory accounts’. We followed the process described by Pearson et al (2015) to integrate these CMOCs into “the most economical expression of Context-Mechanism-Outcome configurations”. We have expressed these as Consolidated CMOCs to express the main programme theories that provide insight into how Nutrition Skills for Life works in supporting socioeconomically deprived groups to have a healthy diet. These are,

- understanding community needs,
- consistent nutrition messages,
- knowledgeable skilled practitioners and
- practicing new skills.

When determining the Consolidated CMOCs (following Pearson et al’s (36) method), first, a novel CMOCs was identified (depicted in blue font). CMOCs that were not novel and therefore similar to this CMOC were grouped together and expressed as a Consolidated CMOC. Important refinements to the novel CMOC are added in colour to show where the refinement originated. We use if – then- because statements in the Consolidated CMOCs to clearly show the Context (If), Outcome (then) and Mechanism (because)

**Understanding community needs – consolidated CMOCs 1-4**

| **Consolidated CMOC 1**    **If** all organisations supporting vulnerable groups to improve eating patterns and nutritional status (C) fully understand the factors that impact on people’s eating patterns and work collaboratively (C), **then** nutritional interventions are more likely to meet individual/ community needs and circumstances (O) resulting in improved nutritional health and wellbeing (O), **because** existing level of knowledge, beliefs and priorities that influence decision making and food choice are acknowledged and more appropriately tailored, practical, trusted advice and support is provided (M).  **Organisations and practitioners understand community needs- consolidated by outcome**  **CMOCs that contribute towards positive outcome i.e., CNIs that meet identified needs and improve dietary intakes 10d, 11a, 14c, 21d, 22c, 32c**  **10d** If the cultural context of communities is fully understood and cultural stereotypes contested (C) then the health needs of communities are more likely to be identified (O) and health improvement interventions designed to meet these needs (O).  [mechanism unclear]  **11a** If practical food skills interventions are designed with knowledge of the unthinking (more habitual) processes involved (materials, meaning, competence) in food choices (C) a deeper, more nuanced understanding of the complex array of factors that influence decision making and food choice (M) can better support families towards greater consumption of un- or minimally processed foods (O) and prevent/ reduce the chance of widening inequalities (O)  **14c If all organisations supporting heroin users to improve eating patterns and nutritional status (C) fully understand the complex array of factors that can negatively impact on users eating patterns and work collaboratively (C), nutritional interventions are more likely to be appropriately tailored to meet individual needs and circumstances (M) and result in improved nutritional intake (O).**  **21d** If those supporting women to manage weight post natally understand the barriers women face (C) such as stress, fatigue, lack of time, their level of existing nutrition knowledge and any ‘ mistaken or false beliefs’ (M) then more tailored, practical healthy eating advice and support can be provided (O) to address the knowledge- attitude gap and facilitate dietary behaviour change (O)  **22c** If primary care clinicians recognise factors that impact on older peoples’ risk of malnutrition such as the social circumstances, cognition, physical health and functioning (C) interventions are delivered by trusted staff and tailored to individual need (M) which can contribute to reducing malnutrition related morbidity and mortality (O).  **32c** When planning a community food initiative in an area of SED consultation and innovation with community members, stakeholders and sponsors at the planning stage (C) can build local partnerships and reshape health priorities (M) ?? how- maybe need to expand here?? to ensure initiatives foster conditions (what conditions) necessary for good health and wellbeing (O)  Partial CMO  **Consolidated CMOC 2**  **If** interventions provide information about healthy food without engaging with the unequal contexts of everyday life that shape healthy food norms, including structural issues (C) **then** interventions are likely to have limited impact (O) **because** of poor understanding of factors impacting on people’s eating patterns and missed opportunities to counteract identified issues (M).  **If organisations and practitioners fail to understand community needs this results in interventions having limited impact - consolidate by outcome**  **CMOCs that contributed to negative outcome 11b, 11f, 11g, 26h, 35a**  **11b** **If practical cooking skills interventions provide information about healthy food or cooking without engaging with, and pushing to the fore, the unequal contexts of everyday life that shape healthy food norms (C) then the non-conscious elements which shape our easy and unthinking actions [money as a stressful constraint, suitability of dining table and greater effort needed to eat if table less easy to eat at and more likely to eat in front of TV] will be missed (M) and interventions are likely to have limited impact (O)**  **11f** If the unequal circumstances in which people ‘choose’ to cook/eat are downplayed (C) then practical aspects (materials, meanings and competencies) than impact on people’s lives/health/food choices [a complex web of mostly unthinking elements] are misunderstood (M) and the needs of those on low income will not be met (O)  **11g** If education and awareness about everyday cooking practices focus on individuals without engaging with context [impact of socioeconomic deprivation on materials, meaning and competence of our lives and the types of food prepared with little conscious attention] (C), there is poor understanding of factors impacting people’s eating and cooking practices (M) then this risks programmes fail to meet participants’ needs, widening inequalities in health and ‘victim blaming’ those for whom the odds are stacked against (O)  **26h** If HCPs caring for young mothers post-partum focus solely on the needs of the baby [e.g. weaning] (C) then the missed opportunity to discuss the mothers health and wellbeing (M) can contribute towards poorer nutrition and health outcomes for both mother and baby (O).  **35a** If projects that aim to tackle food poverty (C) fail to consider wider structural issues such as decline in local shops or poor transport provision (M) ways to counteract these issues will be missed (M) resulting on only modest short-term gains in terms of health-related benefits (O).  **Consolidated CMOC 3**  **If** staff planning and delivering community nutrition interventions with vulnerable community groups encourage discussion and interaction with community members (C) **then** this can maximise engagement, ensure provision is culturally appropriate, increase self-value, self-efficacy, general confidence, resilience and ability to mitigate some of the debilitating aspects of food poverty (O) **because** local partnerships are built and relationships of trust are established which instil trust and confidence in others to share advice and life experiences (M).  **Partnerships and relationships with community members – consolidated by mechanism**  **CMOs contributing are 20a, 22c, 23a, 26g, 32c, 39b**  **20a** **If staff delivering community cooking skills courses for people with low literacy levels encourage group discussion and interaction [relaxed, enthusiastic group atmosphere] (C) and instil trust and confidence to share advice and life experiences, and skill acquisition (M) then this can increase self-value, self-efficacy and general confidence (O ) facilitating positive dietary changes without widening inequalities (O).**  **22c** If primary care clinicians recognise factors that impact on older peoples’ risk of malnutrition such as the social circumstances, cognition, physical health and functioning (C) interventions are delivered by trusted staff and tailored to individual need (M) which can contribute to reducing malnutrition related morbidity and mortality (O).  **23a** If food poverty is discussed with young people by skilled youth workers (C) providing them with an opportunity to express and articulate the structural and political dimensions of food poverty and develop an ability to resist stigma (M) then this can mitigate the debilitating aspects of food poverty (O).  **26g** Given the turbulence inherent in some young women's lives (C), relationships of trust with young women needed to be established (M) before work on health behaviours could be prioritised (O).  **32c** When planning a community food initiative in an area of SED consultation and innovation with community members, stakeholders and sponsors at the planning stage (C) can build local partnerships and reshape health priorities (M) ?? how- maybe need to expand here?? to ensure initiatives foster conditions (what conditions) necessary for good health and wellbeing (O)  Partial CMO  **39b** If facilitators of community based healthy lifestyle programmes for people from diverse cultural groups (C) built relationships with community members (M) to ensure the content and approach was culturally appropriate (M) then this can maximise engagement (O).  **Consolidated CMOC 4**  **If** HCPs who lack the belief that they were able to make a difference to people’s diets receive training to improve their communication and behaviour change skills, knowledge and understanding of complex social and cultural determinants impacting upon individual circumstances and address their expressed prejudices related to stereotypes (C) t**hen** this can support them to more effectively and inclusively meet people’s health needs and facilitate dietary behaviour change (O) **because** they critically reflect on their own emotions, attitudes and beliefs in relation to weight and shape, and gain knowledge, behaviour change skills and confidence (M).  **Addressing expressed prejudices and stereotypes – this is more nuanced than CMOC 1. Consolidated by outcome**  **CMOCs contributing are 10d, 11g, 21d, 26c, 29c**  **10d** If the cultural context of communities is fully understood and cultural stereotypes contested (C) then the health needs of communities are more likely to be identified (O) and health improvement interventions designed to meet these needs (O).  [mechanism unclear]  **11g** If education and awareness about everyday cooking practices focus on individuals without engaging with context [impact of socioeconomic deprivation on materials, meaning and competence of our lives and the types of food prepared with little conscious attention] (C), there is poor understanding of factors impacting people’s eating and cooking practices (M) then this risks programmes fail to meet participants’ needs, widening inequalities in health and ‘victim blaming’ those for whom the odds are stacked against (O)  **21d** If those supporting women to manage weight post natally understand the barriers women face (C) such as stress, fatigue, lack of time, their level of existing nutrition knowledge and any ‘ mistaken or false beliefs’ (M) then more tailored, practical healthy eating advice and support can be provided (O) to address the knowledge- attitude gap and facilitate dietary behaviour change (O)  **26c** If HCPs who provide guidance and support on healthy eating for young women during pregnancy (C) critically reflect on their own emotions, attitudes and beliefs in relation to weight and shape (M) and improve their knowledge and understanding of complex social determinants impacting upon individual circumstances (M) then this can support them to effectively discuss weight management and potential body image concerns of young women (O)  **29c** **If HCPs who lacked the belief that they were able to make a difference to women’s behaviours during pregnancy receive training [in motivational interviewing, having healthy conversations, and goal setting] to address their expressed prejudices towards people living with obesity, related to stereotypes (C) then this can improve knowledge, behaviour change skills and confidence (M) to provide weight management support (O).** |
| --- |

***CMOCs contributing to consolidated CMOC 1-4***

| **Paper** | **CMOCs planning/ delivery/impact- positive** | **CMOCs planning/ delivery/impact -negative** |
| --- | --- | --- |
| 10 ^(45)^ | **10d** If the cultural context of communities is fully understood and cultural stereotypes contested (C) then the health needs of communities are more likely to be identified (O) and health improvement interventions designed to meet these needs (O).  [mechanism unclear] |  |
| 11 ^(46)^ | **11a** If practical food skills interventions are designed with knowledge of the unthinking (more habitual) processes involved (materials, meaning, competence) in food choices (C) a deeper, more nuanced understanding of the complex array of factors that influence decision making and food choice (M) can better support families towards greater consumption of un- or minimally processed foods (O) and prevent/ reduce the chance of widening inequalities (O) | **11b** If practical cooking skills interventions provide information about healthy food or cooking without engaging with, and pushing to the fore, the unequal contexts of everyday life which shape healthy food norms (C) then the non-conscious elements which shape our easy and unthinking actions [money as a stressful constraint, suitability of dining table and greater effort needed to eat if table less easy to eat at and more likely to eat in front of TV] will be missed (M) and interventions are likely to have limited impact (O) |
|  |  | **11f** If the unequal circumstances in which people ‘choose’ to cook/eat are downplayed (C) then practical aspects (materials, meanings and competencies) than impact on people’s lives/health/food choices [a complex web of mostly unthinking elements] are misunderstood (M) and the needs of those on low income will not be met (O) |
|  |  | **11g** If education and awareness about everyday cooking practices focus on individuals without engaging with context [impact of socioeconomic deprivation on materials, meaning and competence of our lives and the types of food prepared with little conscious attention] (C), there is poor understanding of factors impacting people’s eating and cooking practices (M) then this risks programmes fail to meet participants’ needs, widening inequalities in health and ‘victim blaming’ those for whom the odds are stacked against (O) |
| 14 ^(49)^ | **14c** If all organisations supporting heroin users to improve eating patterns and nutritional status (C) fully understand the complex array of factors that can negatively impact on users eating patterns and work collaboratively (C), nutritional interventions are more likely to be appropriately tailored to meet individual needs and circumstances (M) and result in improved nutritional intake (O). |  |
| 20 ^(55)^ | **20a** If staff delivering community cooking skills courses for people with low literacy levels encourage group discussion and interaction [relaxed, enthusiastic group atmosphere] (C) and instil trust and confidence to share advice and life experiences, and skill acquisition (M) then this can increase self-value, self-efficacy and general confidence (O ) facilitating positive dietary changes without widening inequalities (O). |  |
| 21 ^(56)^ | **21d** If those supporting women to manage weight post natally understand the barriers women face (C) such as stress, fatigue, lack of time, their level of existing nutrition knowledge and any ‘ mistaken or false beliefs’ (M) then more tailored, practical healthy eating advice and support can be provided (O) to address the knowledge- attitude gap and facilitate dietary behaviour change (O) |  |
| 22 ^(57)^ | **22c** If primary care clinicians recognise factors that impact on older peoples’ risk of malnutrition such as the social circumstances, cognition, physical health and functioning (C) interventions are delivered by trusted staff and tailored to individual need (M) which can contribute to reducing malnutrition related morbidity and mortality (O). |  |
| 23 ^(58)^ | **23a** If food poverty is discussed with young people by skilled youth workers (C) providing them with an opportunity to express and articulate the structural and political dimensions of food poverty and develop an ability to resist stigma (M) then this can mitigate the debilitating aspects of food poverty (O). |  |
| 26 ^(61)^ | **26c** If HCPs who provide guidance and support on healthy eating for young women during pregnancy (C) critically reflect on their own emotions, attitudes and beliefs in relation to weight and shape (M) and improve their knowledge and understanding of complex social determinants impacting upon individual circumstances (M) then this can support them to effectively discuss weight management and potential body image concerns of young women (O) | **26h** If HCPs caring for young mothers post-partum focus solely on the needs of the baby [e.g. weaning] (C) then the missed opportunity to discuss the mothers health and wellbeing (M) can contribute towards poorer nutrition and health outcomes for both mother and baby (O). |
|  | **26g** Given the turbulence inherent in some young women's lives (C), relationships of trust with young women needed to be established (M) before work on health behaviours could be prioritised (O). |  |
| 29 ^(64)^ | **29c** If HCPs who lacked the belief that they were able to make a difference to women’s behaviours during pregnancy receive training [in motivational interviewing, having healthy conversations, and goal setting] to address their expressed prejudices towards obese populations, related to stereotypes (C) then this can improve knowledge, behaviour change skills and confidence (M) to provide weight management support (O). |  |
| 32 ^(67)^ | **32c** When planning a community food initiative in an area of SED consultation and innovation with community members, stakeholders and sponsors at the planning stage (C) can build local partnerships and reshape health priorities (M) ?? how- maybe need to expand here?? to ensure initiatives foster conditions (what conditions) necessary for good health and wellbeing (O)  Partial CMO |  |
| 35 ^(70)^ |  | **35a** If projects that aim to tackle food poverty (C) fail to consider wider structural issues such as decline in local shops or poor transport provision (M) ways to counteract these issues will be missed (M) resulting on only modest short-term gains in terms of health-related benefits (O). |
| 39 ^(74)^ | **39b** If facilitators of community based healthy lifestyle programmes for people from diverse cultural groups (C) built relationships with community members (M) to ensure the content and approach was culturally appropriate (M) then this can to maximise engagement (O). |  |

**Consistent nutrition messages - consolidated CMOCs 5 - 8**

| **Consolidated CMOC 5**  **If** people from vulnerable groups have incomplete knowledge of nutrition and do not receive reliable information and advice from trusted sources, despite high desire to provide a healthy diet for themselves or their family, t**hen** this can lead to poor adherence to dietary recommendations and greater risk of poor nutritional intake, b**ecause** of confusion, mistaken beliefs and commonly held perceptions about how and why to make healthier choices and seeking information from informal sources  **Confusion and mistaken beliefs can result in poor adherence to dietary recommendations and poor dietary intake- consolidated by outcome.**  **CMOCs contributing are** **2b, 2c, 3c, 5a, 5c, 22d, 22e, 37a**  **2b** **When pregnant women with incomplete knowledge about the importance of nutrition and vitamin supplementation during pregnancy seek information on the internet, some of which is not supported by NICE (C), then this can lead to mistaken beliefs [such as ‘eat for two’] (M) and poor adherence to dietary recommendations during pregnancy (O).**  **2c** If women were not advised by an HCP on the importance of taking folic acid, vitamin D and on improving nutrition before & during pregnancy (C) then incomplete knowledge or mistaken beliefs [from internet sources] (M) resulting in poor compliance with supplementation, increasing the risk of nutritional deficiencies (O).  **3c** If pregnant women are unaware of the risks associated with obesity and excess gestational weight gain (GWG) (C) they may consider weight gain as ‘inevitable’, relaxing previous dietary restrictions (M) then this could increase their risk of excess GWG and post-partum weight retention (O).  **5a** If healthcare professionals do not offer parents enough detailed advice about the timing of introduction of solid foods to infants (C) mothers may mistake normal developmental behaviour (chewing fists, waking at night) for signs of hunger (M) then introduce solids earlier than recommended (O).  **5c** When parents lacked knowledge of what constitutes healthy eating, despite high desire to provide a healthy diet for their family (C) their confusion about how/why to make healthier choices (M) resulted in inadvertently including HFSS foods in children’s diets (O)  **22d** If a combination of commonly held perceptions [such as being thin is healthy and ‘snacking’ is unhealthy] and a lack of reliable information about what a healthy diet should consist of in later life [and how to manage a low appetite] (C) then there is an increased risk of inappropriate dietary provision for older people who are at greater risk of malnutrition (M) which can lead to poor nutritional intake and weight loss (O).  **22e** If older people who are at greater risk of malnutrition (C), hold a combination of commonly held perceptions such as being thin is healthy and ‘snacking’ is unhealthy (M – reasoning), lack of reliable information about what a healthy diet should consist of in later life and low appetite (M- resource) can lead to poor nutritional intake and weight loss (O).  **37a** If younger first-time mothers have poorer knowledge of the current guidelines for weaning (C) and seek information from informal sources such as family, particularly the previous generation (M) then this can lead to inappropriate early weaning (O).  **Consolidated CMOC 6**  **Dispelling myths and reducing confusion by cascading evidence-based messages -consolidated by outcome**  **CMOCs contributing are 3b, 21d, 22a, 22b,**  **If** healthcare professionals caring for vulnerable people have knowledge and understanding of people’s nutritional needs and their level of existing knowledge and/or mistaken or false beliefs **then** this can ensure people receive correct information, do not receive conflicting messages, are not advised to follow unsafe practices and knowledge-attitude gaps are addressed **because** HCPs can adapt dietary advice to meet individual’s needs, correct nutrition information is available, and confusion from conflicting messages is avoided  Conversely, If HCPs have insufficient knowledge and understanding of people’s nutritional needs **then** this can lead to unsafe practice and missed opportunities to facilitate behaviour change and address knowledge-attitude gaps **because** correct nutrition information is unavailable and people feel unsupported.  **3b** Pregnant women in this study, motivated to make dietary changes (C) reported that insufficient knowledge and a lack of support from healthcare professionals (M) limited the nutrition information available (O)  **21d** If those supporting women to manage weight post natally understand the barriers women face (C) such as stress, fatigue, lack of time, their level of existing nutrition knowledge and any ‘ mistaken or false beliefs’ (M) then more tailored, practical healthy eating advice and support can be provided (O) to address the knowledge- attitude gap and facilitate dietary behaviour change (O)  **22a** If healthcare professionals have knowledge and understanding of the nutritional needs of older people who are at risk of malnutrition (C) older people receive correct information, do not receive conflicting messages (M) are not advised to follow restricting diets that are potentially harmful (O)  **22b** **If health professionals caring for older people who are at risk of malnutrition (C), have good knowledge and understanding [through education] of how to deliver dietary advice adapted to older people’s needs (M-resource) this can ensure older people receive correct information, do not receive conflicting messages and are not advised to follow restricting diets that are potentially harmful (O).**  **Consolidated CMOC 7**    **If** unhealthy foods were provided alongside healthy options in retail and public sector settings and were more affordable **then** conflicting messages had the potential to undermine efforts to encourage healthier food consumption behaviour **because** of confusion due to the food provided not aligning with nutritional guidelines, unhealthy options being considered ‘a treat’ and perception that healthy foods are less affordable.  **Conversely,**  **If** healthy foods were cooked alongside staff during on-site cooking sessions to cook full meals and healthy foods were more affordable **then** this can minimise conflicting messages that undermine efforts to encourage healthier food consumption behaviour **because** food provided aligns with nutritional guidelines, nutrition education sessions are embedded and the perception that healthy foods were unaffordable is reduced.  **Food provision in settings aligns with key messages and dietary guidelines – consolidated by outcome**  **CMOCs contributing are 19b,19d, 27b, 36e**  **19b** If HAF participants were able to cook alongside trained staff during on-site cooking sessions to cook full meals (C), then nutrition education was embedded and any possible discontinuity between the NE messages and the quality and variety of food provided was minimised (M) avoiding conflicting and confusing messages (O).  **19d** If providers of the free Holiday Activities and Food (HAF) programme for families and children on a low income (C), accepted food donations from charities and food retailers such as doughnuts, cakes, pastries and sweetened fizzy drinks, that were not in line with healthy eating guidelines (M-resource) but were given to children as considered a ‘treat’ (M-reasoning) then this had the potential to undermine the NE sessions and conveying conflicting messages for programme participants (O).  **27b If businesses that held a healthier catering award displayed and sold healthy options alongside extremely unhealthy cakes and snacks (C), conflicting messages whereby foods on offer did not align with healthy eating guidelines (M) undermined efforts to encourage healthier food consumption behaviour (O)**  **36e** If public health interventions to promote home cooking are supported by measures to increase the affordability of basic ingredients (C) to reduce the perception that fruit and vegetable are unaffordable (M) then this can increase F & V purchase and consumption (O)  **Consolidated CMOC 8**  **If** nutrition education and practical cooking skills development opportunities are provided at life stages when people are most receptive (e.g., early parenthood, young adults, people with long term conditions) including digital technology and social media interventions/resources **then** this can facilitate dietary behaviour change, provide ‘the tools needed for a healthy lifelong relationship with food’, encourage adoption of healthy cooking habits into daily routines and manage food waste well which can influence food spending, **because of** increased motivation to feed their family a nutritious diet, improvements in food literacy - learning about nutrition and the importance of a balanced diet for health, learning practical food preparation skills and new recipes, understanding food labels, portion sizes and ways to encourage children to eat well  **Opportunities to learn - nutrition education / healthy eating support and access to consistent nutrition messages – consolidated by mechanism which includes access to nutrition information / consistent nutrition messages**  **CMOCs contributing are 5d, 12a, 15c, 18d, 20b, 24c, 34c, 39a**  **5d** **If nutrition education and practical cooking skills development opportunities are provided during early parenthood, when parents are most receptive (C) this can increase their knowledge, skills [physical and psychological capability] and potentially motivation (M) to feed their family a nutritious diet (O)**  **12a** When parents of preschool children were questioned about the types of healthy eating support they would find helpful (C), then less well educated parents (C) wanted to learn more about; what a ‘Healthy diet’ means, how to cook and prepare healthy food, how to understand food labels, budgeting for food, examples of healthy food and snacks for children, appropriate portion sizes for children and ways to encourage children to eat well (M) to support themselves and their children to have a healthy diet (O).  **15c** If an Internet-based diet application for adults in a workplace setting (C) included desirable features such as recipes, interactivity, nutritional information, shopping tips, cost-saving information, and a companion smartphone app (M) then this could facilitate dietary behaviour change (O).  **18d** If people have the opportunity to attend a 6 week cooking course] (C) improvements in nutrition knowledge and practical use of food label components; understanding correct portion sizes and awareness of the importance of a balanced diet for health (M) can result in more deliberate and controlled food intake, which is essential in regulating over-eating and managing food waste well, which in turn influences food spending (O).  **20b** If people on a low income attend a 4-10 week cooking course (C), improved knowledge of healthy eating e.g. more aware of health benefits of fruit and veg, health consequences of too much saturated fat, ways to reduce fat and oil and how to read food labels (M) and learning new recipes and ways to cook from scratch (M) can increase the likelihood that people can manage long term conditions such as obesity or diabetes (O)  **24c** If public health programmes want to increase the number of people who learn to cook healthy meals and to use their skills often (C) then using digital technology and social media to provide shopping list generators, food preparation teaching videos, and nutritional information (M) could enable people to adopt cooking habits as part of their daily routine and improve their health and wellbeing (O).  **34c** If public health and home economics education and training are provided (C) to develop young people’s technical cooking skills, broader general food skills, and food literacy (M) then this can provide ‘the tools needed for a healthy lifelong relationship with food’ (O) and grow capacity for healthy cooking at home (O). |
| --- |

***CMOCs contributing to consolidated CMOC 5 - 8***

| **Paper** | **CMOCs with nutrition knowledge** | **CMOCs Lack of nutrition knowledge/lack of consistent messages** |  |
| --- | --- | --- | --- |
| 2 ^(37)^ |  | **2b** When pregnant women with incomplete knowledge about the importance of nutrition and vitamin supplementation during pregnancy seek information on the internet, some of which is not supported by NICE (C), then this can lead to mistaken beliefs [such as ‘eat for two’] (M) and poor adherence to dietary recommendations during pregnancy (O). |  |
|  |  | **2c** If women were not advised by an HCP on the importance of taking folic acid, vitamin D and on improving nutrition before & during pregnancy (C) then incomplete knowledge or mistaken beliefs [from internet sources] (M) resulting in poor compliance with supplementation, increasing the risk of nutritional deficiencies (O). |  |
| 3 ^(38)^ |  | **3b** Pregnant women in this study, motivated to make dietary changes (C) reported that insufficient knowledge and a lack of support from healthcare professionals (M) limited the nutrition information available (O) |  |
|  |  | **3c** If pregnant women are unaware of the risks associated with obesity and excess gestational weight gain (GWG) (C) they may consider weight gain as ‘inevitable’, relaxing previous dietary restrictions (M) then this could increase their risk of excess GWG and post-partum weight retention (O). |  |
|  | **3f** A digital health intervention (such as an App) about nutrition and diet during pregnancy (C) could strengthen the midwife-patient relationship by increasing the midwife’s knowledge and self-confidence to give personalised advice (M) and enable women to access information relevant to their specific dietary circumstances (O). |  |  |
| 5 ^(40)^ | **5d** If nutrition education and practical cooking skills development opportunities are provided during early parenthood, when parents are most receptive (C) and increasing their knowledge, skills [physical and psychological capability] and potentially motivation (M) to feed their family a nutritious diet (O) | **5a** If healthcare professionals do not offer parents enough detailed advice about the timing of introduction of solid foods to infants (C) mothers may mistake normal developmental behaviour (chewing fists, waking at night) for signs of hunger (M) then introduce solids earlier than recommended (O). |  |
|  |  | **5c** When parents lacked knowledge of what constitutes healthy eating, despite high desire to provide a healthy diet for their family (C) their confusion about how/why to make healthier choices (M) resulted in inadvertently including HFSS foods in children’s diets (O) |  |
| 12 ^(47)^ | **12a** When parents of preschool children were questioned about the types of healthy eating support they would find helpful (C), then less well educated parents wanted to learn more about; what a ‘Healthy diet’ means, how to cook and prepare healthy food, how to understand food labels, budgeting for food, examples of healthy food and snacks for children, appropriate portion sizes for children and ways to encourage children to eat well (M) to support themselves and their children to have a healthy diet (O). |  |  |
| 15 ^(50)^ | **15c** If an Internet-based diet application for adults in a workplace setting (C) included desirable features such as recipes, interactivity, nutritional information, shopping tips, cost-saving information, and a companion smartphone app (M) then this could facilitate dietary behaviour change (O). |  |  |
| 18 ^(53)^ | **18d** If people have the opportunity to attend a 6 week cooking course] (C) improvements in nutrition knowledge and practical use of food label components; understanding correct portion sizes and awareness of the importance of a balanced diet for health (M) can result in more deliberate and controlled food intake, which is essential in regulating over-eating and managing food waste well, which in turn influences food spending (O). |  |  |
| 19 ^(54)^ | **19b** If HAF participants were able to cook alongside trained staff during on-site cooking sessions to cook full meals (C), then nutrition education was embedded and any possible discontinuity between the NE messages and the quality and variety of food provided was minimised (M) avoiding conflicting and confusing messages (O). |  |  |
|  |  | **19d** If providers of the free Holiday Activities and Food (HAF) programme for families and children on a low income (C), accepted food donations from charities and food retailers such as doughnuts, cakes, pastries and sweetened fizzy drinks, that were not in line with healthy eating guidelines (M-resource) but were given to children as considered a ‘treat’ (M-reasoning) then this had potential to undermine the NE sessions and conveying conflicting messages for programme participants (O). |  |
| 20 ^(55)^ | **20b** If people on a low income attend a 4-10 week cooking course (C), improved knowledge of healthy eating e.g. more aware of health benefits of fruit and veg, health consequences of too much saturated fat, ways to reduce fat and oil and how to read food labels (O) and learning new recipes and ways to cook from scratch (O) can increase the likelihood that people can manage long term conditions such as obesity or diabetes (O) Not sure of mechanism |  |  |
| 21 ^(56)^ | **21d** If those supporting women to manage weight post natally understand the barriers women face (C) such as stress, fatigue, lack of time, their level of existing nutrition knowledge and any ‘ mistaken or false beliefs’ (M) then more tailored, practical healthy eating advice and support can be provided (O) to address the knowledge- attitude gap and facilitate dietary behaviour change (O) |  |  |
| 22 ^(57)^ | **22a** If healthcare professionals have knowledge and understanding of the nutritional needs of older people who are at risk of malnutrition (C) older people receive correct information, do not receive conflicting messages (M) are not advised to follow restricting diets that are potentially harmful (O) | **22d** If a combination of commonly held perceptions [such as being thin is healthy and ‘snacking’ is unhealthy] and a lack of reliable information about what a healthy diet should consist of in later life [and how to manage a low appetite] (C) then there is an increased risk of inappropriate dietary provision for older people who are at greater risk of malnutrition (M) which can lead to poor nutritional intake and weight loss (O). |  |
|  | **22b** If health professionals caring for older people who are at risk of malnutrition (C), have good knowledge and understanding [through education] of how to deliver dietary advice adapted to older people’s needs (M-resource) this can ensure older people receive correct information, do not receive conflicting messages and are not advised to follow restricting diets that are potentially harmful (O). | **22e** If older people who are at greater risk of malnutrition (C), hold a combination of commonly held perceptions such as being thin is healthy and ‘snacking’ is unhealthy (M – reasoning), lack of reliable information about what a healthy diet should consist of in later life and low appetite (M- resource) can lead to poor nutritional intake and weight loss (O). |  |
| 24 ^(59)^ | **24c** If public health programmes want to increase the number of people who learn to cook healthy meals and to use their skills often (C) then using digital technology and social media to provide shopping list generators, food preparation teaching videos, and nutritional information (M) could enable people to adopt cooking habits as part of their daily routine and improve their health and wellbeing (O). |  |  |
| 27 ^(62)^ |  | **27b** If businesses that held a healthier catering award displayed and sold healthy options alongside extremely unhealthy cakes and snacks (C), conflicting messages whereby foods on offer did not align with healthy eating guidelines (M) undermined efforts to encourage healthier food consumption behaviour (O) |  |
| 34 ^(69)^ | **34c** If public health and home economics education and training are provided (C) to develop young people’s technical cooking skills, broader general food skills, and food literacy (M) then this can provide ‘the tools needed for a healthy lifelong relationship with food’ (O) and grow capacity for healthy cooking at home (O). |  |  |
| 36 ^(71)^ |  | **36e** If public health interventions to promote home cooking are supported by measures to increase the affordability of basic ingredients (C) to reduce the perception that fruit and vegetable are unaffordable (M) then this can increase F & V purchase and consumption (O) |  |
| 37 ^(72)^ |  | **37a** If younger first-time mothers have poorer knowledge of the current guidelines for weaning (C) and seek information from informal sources such as family, particularly the previous generation (M) then this can lead to inappropriate early weaning (O). | |

**Knowledgeable , skilled, confident practitioners/facilitators – Consolidated CMOCs 9-12**

| **Consolidated CMOC 9**  **If** health care practitioners receive training and education in nutrition, behaviour change techniques such as motivational interviewing, having healthy conversations and goal setting **then** they are more likely to feel better equipped to discuss eating habits, provide dietary advice and support behaviour change for vulnerable community groups **because** they are more confident in their nutrition knowledge, have greater self-confidence and self-efficacy to raise sensitive topics , empowered with skills to take a structured approach to conversations and motivated to discuss health behaviours.  **Staff confidence – sorted by mechanism**  **CMOCs contributing are 3d, 13d, 26a, 26b, 26e, 29c, 44c**  **3d** If dietitians with pregnancy nutrition expertise, delivered in-depth nutrition information as part of the undergraduate curriculum (C) this would enhance nutrition knowledge and empower midwives (M) to support behaviour change (O).  **13d** If midwives are provided with training in behaviour change techniques such as MI (C) this increased their confidence to use evidence-based MI skills in practice to facilitate behaviour change (M) resulting in women feeling supported to modify their dietary behaviours (O).  **26a** If HCPs caring for young women during and after pregnancy (C) had confidence in their nutrition knowledge and training in behaviour change techniques (M) rather than relying on tacit knowledge and common sense (M) then they are more likely to feel better equipped to discuss eating habits (O)  **26b** If HCPs caring for young women during and after pregnancy(C) had confidence in their nutrition knowledge, and training in behaviour change techniques, and were empowered with skills to take a structured approach to conversations (M) then they are more likely to feel better equipped to discuss eating habits rather than relying on tacit knowledge and common sense (O) when supporting women to adopt healthy behaviours (O).  **26e** **If healthcare professionals [midwives and HVs] were supported with education and training to increase knowledge and communication skills (C) then they were more confident and motivated to discuss health behaviours(M) to improve nutrition and health outcomes for young mothers (O).**  **29c** If HCPs who lacked the belief that they were able to make a difference to women’s behaviours during pregnancy receive training [in motivational interviewing, having healthy conversations, and goal setting] express prejudices towards obese populations, related to stereotypes (C) then this can improve knowledge, behaviour change skills and confidence (M) to provide weight management support (O).  **44c** If training for practitioners working with parents of pre-school children around overweight and obesity focusses on how to have compassionate, non-judgemental conversations (C) giving staff greater self-confidence and self-efficacy to raise the topic (M) then this can increase the number of healthy conversations and support provided for families (O).  **Consolidated CMOC 10**  **If** healthcare practitioners caring for people living with obesity are motivated but lack the confidence to raise the topic of weight **then** they might avoid discussing eating habits **because** they are anxious and fearful of coming across as judgemental and lacking specialist knowledge of how to address eating, offending and causing upset, and negatively impacting their relationship with women.  **Conversely,**  **If** training is provided to address knowledge gaps for health care practitioners who care for people living with obesity **then** this can increase the number of healthy conversations and improve care and support provided for vulnerable groups **because** this can give staff greater confidence and self-efficacy to raise the topic, alleviate anxiety about discussing weight, reduce fear of coming across as judgemental and lacking specialist knowledge of how to address eating and could positively impact their relationship with women.  **Alleviating fear of causing offence – consolidated by mechanism**  **CMOCs contributing are 26d, 29a, 44c**  **26d** **If HCPs caring for young pregnant women who are living with obesity (C) are anxious about discussing weight, fearful of coming across as judgemental and lacking specialist knowledge of how to address eating (M) then they might avoid discussing eating habits (O).**  **29a** If HCPs are motivated, but lack confidence to sensitively raise the topic of maternal obesity with women during pregnancy attend training to address knowledge gaps (C) then this could alleviate their fears of offending, causing upset, negatively impact their relationship with women (M) and improve maternal care (O).  **44c** If training for practitioners working with parents of pre-school children around overweight and obesity focusses on how to have compassionate, non-judgemental conversations (C) giving staff greater self-confidence and self-efficacy to raise the topic (M) then this can increase the number of healthy conversations and support provided for families (O).  **Consolidated CMOC 11**  **If** families are supported with positive, practical messages about healthy eating, delivered by confident knowledgeable and skilled professionals who use motivational interviewing and encourage people to set their own SMART goals for dietary change that they are able to achieve **then** this supports behaviour change for dietary improvement in the early years **because** the advice is regarded as reliable and trustworthy, raises people’s confidence and self-efficacy to make incremental dietary changes, gives a sense of ownership over the goals and accomplishment and pride when goals were met.  **Conversely**  **If** families do not receive this support **then** behaviour change is not supported **because** of their incomplete knowledge or mistaken beliefs from informal sources and insufficient knowledge and support from HCPs  **Impact of staff nutrition knowledge, BCT skills and confidence on facilitating others to overcome challenges and change behaviour- contributing to optimal nutritional care (positive). Consolidated by outcome**  **Contributing CMOCs are 2a, 3a, 3e, 13a, 13b, 13c, 37b**  **2a** When nutrition, vitamin supplementation and Healthy Start information [during pregnancy] is provided by ante natal nurses or pharmacists (C)[via motivational interviewing] rather than the internet (?M) then this is regarded as more reliable by women (M) in supporting behaviour change (O)  **3a If women receive positive, practical messages about healthy eating during pregnancy (focussing on what they can eat rather than want they can’t) (C) delivered by confident, knowledgeable professionals (M) then this can raise women’s awareness, expectations, confidence and intention to change (O) enabling them to overcome challenges such as nausea, vomiting, anxiety about weight and lack of time (O).**  **3e** If midwives are skilled in using behaviour change techniques such as goal setting and self-monitoring (C) to tailor advice towards women’s  expectations, confidence and behavioural intention (M) then this can increase engagement in lifestyle behaviour change during pregnancy(O).  **13a** If midwives delivered brief interventions for women during pregnancy (C) using motivation interviewing and goal setting strategies (M), accompanied by a goals card which acted as a reminder and prompt to achieve their goals [such as swapping chocolate and crisps with fruit or nuts, walking children to school instead of taking the car] giving women a sense of accomplishment and pride (M) and confidence (for some) (M) then this often resulted in them aiming for further improvements to their lifestyle beyond the initial goals set (O).  **13b** When pregnant women were supported [by HCPs using MI skills] to set their own goals for dietary change (C) they felt a sense of ownership over the goals and changes were self-driven, supporting their sense of autonomy (M) in pursuing healthy lifestyle in pregnancy (O) giving women a sense of accomplishment and pride (M) and confidence (for some) (M) then this often resulted in them aiming for further improvements to their lifestyle beyond the initial goals set (O).  **13c** If pregnant women are encouraged to set their own, SMART goals for dietary change that they were able to achieve (C) this built on their sense of confidence and competence [self-efficacy] in their ability (M) to continue making small, realistic dietary changes for health improvement (O).  **37b** When a first-time mother’s position on the weaning guidelines and existing weaning knowledge are acknowledged (C) greater confidence and trust in the advice given by the HV (M) can mean that weaning information is more likely to be well received and adhered to (O).  **Impact of lack of staff nutrition knowledge, BCT skills and confidence – missed opportunity to facilitate others to overcome challenges and change behaviour- not contributing to optimal nutritional care (negative). Consolidated by outcome**  **Contributing CMOCs are 2c,3b**  **2c** **If women were not advised by an HCP on the importance of taking folic acid, vitamin D and on improving nutrition before & during pregnancy (C) then incomplete knowledge or mistaken beliefs [from internet sources] (M) resulting in poor compliance with supplementation, increasing the risk of nutritional deficiencies (O).**  **3b** Pregnant women in this study, motivated to make dietary changes (C) reported that insufficient knowledge and a lack of support from healthcare professionals (M) limited the nutrition information available (O)  **Consolidated CMOC 12**  If staff caring for older adults receive training to provide knowledge and understanding of the nutritional needs of older people who are at risk of undernutrition, are alert for signs of malnutrition and are able to offer older adults and their relatives advice and support **then** this can contribute towards the provision of good nutritional care **because** commonly held perceptions are addressed, confusion over conflicting messages is avoided, **dietary advice can be appropriately adapted to meet individuals’ needs and** relatives can proactively contribute.  **Similar to CMOC 11, however, the context is improved nutritional care for older adults- consolidated by outcome**  **CMOCs contributing are 16c, 22a, 22b, 22e, 33c**  **16c** If community nurses visiting older people in their own homes are alert for signs of malnutrition and are able to offer help, advice and support to relatives (C) then relatives and carers can be proactive in meeting that person’s dietary needs (M) preventing health decline due to poor sustenance (O)  **22a** **If healthcare professionals have knowledge and understanding of the nutritional needs of older people who are at risk of malnutrition (C) older people receive correct information, do not receive conflicting messages (M) are not advised to follow restricting diets that are potentially harmful (O)**  **22b** If health professionals caring for older people who are at risk of malnutrition (C), have good knowledge and understanding [through education] of how to deliver dietary advice adapted to older people’s needs (M-resource) this can ensure older people receive correct information, do not receive conflicting messages and are not advised to follow restricting diets that are potentially harmful (O).  **22e** If older people who are at greater risk of malnutrition (C), hold a combination of commonly held perceptions such as being thin is healthy and ‘snacking’ is unhealthy (M – reasoning), lack of reliable information about what a healthy diet should consist of in later life and low appetite (M- resource) can lead to poor nutritional intake and weight loss (O).  **33c** If care home staff receive appropriate training on nutrition, including special dietary needs (C), to overcome the dilemma of wanting to provide a ‘healthy diet’ whilst also responding to individual autonomy, needs and choice (M) then this can contribute towards the provision of good nutritional care (O). |
| --- |

***CMOCs contributing to consolidated CMOCs 9-12***

| **Paper** | **CMOCs HCPs/Community group facilitators** | **CMOCs The public/group participants** |
| --- | --- | --- |
| **2** ^(37)^ |  | **2a** When nutrition, vitamin supplementation and Healthy Start information [during pregnancy] is provided by ante natal nurses or pharmacists (C)[via motivational interviewing] rather than the internet (?M) then this is regarded as more reliable by women (M) in supporting behaviour change (O) |
|  |  | **2c** If women were not advised by an HCP on the importance of taking folic acid, vitamin D and on improving nutrition before & during pregnancy (C) then incomplete knowledge or mistaken beliefs [from internet sources] (M) resulting in poor compliance with supplementation, increasing the risk of nutritional deficiencies (O). |
| **3** ^(38)^ | **3d** If dietitians with pregnancy nutrition expertise, delivered in-depth nutrition information as part of the undergraduate curriculum (C) this would enhance nutrition knowledge and empower midwives (M) to support behaviour change (O). | **3a** If women receive positive, practical messages about healthy eating during pregnancy (focussing on what they can eat rather than want they can’t) (C) delivered by confident, knowledgeable professionals (M) then this can raise women’s awareness, expectations, confidence and intention to change (O) enabling them to overcome challenges such as nausea, vomiting, anxiety about weight and lack of time (O). |
|  | **3e** If midwives are skilled in using behaviour change techniques such as goal setting and self-monitoring (C) to tailor advice towards women’s  expectations, confidence and behavioural intention (M) then this can increase engagement in lifestyle behaviour change during pregnancy(O). | **3b** Pregnant women in this study, motivated to make dietary changes (C) reported that insufficient knowledge and a lack of support from healthcare professionals (M) limited the nutrition information available (O) |
| **13** ^(48)^ | **13a** If midwives delivered brief interventions for women during pregnancy (C) using motivation interviewing and goal setting strategies (M), accompanied by a goals card which acted as a reminder and prompt to achieve their goals [such as swapping chocolate and crisps with fruit or nuts, walking children to school instead of taking the car] giving women a sense of accomplishment and pride (M) and confidence (for some) (M) then this often resulted in them aiming for further improvements to their lifestyle beyond the initial goals set (O). | **13b** When pregnant women were supported [by HCPs using MI skills] to set their own goals for dietary change (C) they felt a sense of ownership over the goals and changes were self-driven, supporting their sense of autonomy (M) in pursuing healthy lifestyle in pregnancy (O)  giving women a sense of accomplishment and pride (M) and confidence (for some) (M) then this often resulted in them aiming for further improvements to their lifestyle beyond the initial goals set (O). |
|  | **13d** If midwives are provided with training in behaviour change techniques such as MI (C) this increased their confidence to use evidence-based MI skills in practice to facilitate behaviour change (M) resulting in women feeling supported to modify their dietary behaviours (O). | **13c** If pregnant women are encouraged to set their own, SMART goals for dietary change that they were able to achieve (C) this built on their sense of confidence and competence [self-efficacy] in their ability (M) to continue making small, realistic dietary changes for health improvement (O). |
| **26** ^(61)^ | **26a** If HCPs caring for young women during and after pregnancy (C) had confidence in their nutrition knowledge and training in behaviour change techniques (M) rather than relying on tacit knowledge and common sense (M) then they are more likely to feel better equipped to discuss eating habits (O) |  |
|  | **26b** If HCPs caring for young women during and after pregnancy(C) had confidence in their nutrition knowledge, and training in behaviour change techniques, and were empowered with skills to take a structured approach to conversations (M) then they are more likely to feel better equipped to discuss eating habits rather than relying on tacit knowledge and common sense (O) when supporting women to adopt healthy behaviours (O). |  |
|  | **26d** If HCPs caring for young pregnant women who are living with obesity (C) are anxious about discussing weight, fearful of coming across as judgemental and lacking specialist knowledge of how to address eating (M) then they might avoid discussing eating habits (O) |  |
|  | **26e** If healthcare professionals [midwives and HVs] were supported with education and training to increase knowledge and communication skills (C) then they were more confident and motivated to discuss health behaviours(M) to improve nutrition and health outcomes for young mothers (O). |  |
| **29** ^(64)^ | **29a** If HCPs are motivated, but lack confidence to sensitively raise the topic of maternal obesity with women during pregnancy attend training to address knowledge gaps (C) then this could alleviate their fears of offending, causing upset, negatively impact their relationship with women (M) and improve maternal care (O). |  |
|  | **29c** If HCPs who lacked the belief that they were able to make a difference to women’s behaviours during pregnancy receive training [in motivational interviewing, having healthy conversations, and goal setting] express prejudices towards obese populations, related to stereotypes (C) then this can improve knowledge, behaviour change skills and confidence (M) to provide weight management support (O). |  |
| **44** ^(79)^ | **44c** If training for practitioners working with parents of pre-school children around overweight and obesity focusses on how to have compassionate, non-judgemental conversations (C) giving staff greater self-confidence and self-efficacy to raise the topic (M) then this can increase the number of healthy conversations and support provided for families (O). |  |
| **16** ^(51)^ | **16c** If community nurses visiting older people in their own homes are alert for signs of malnutrition and are able to offer help, advice and support to relatives (C) then relatives and carers can be proactive in meeting that person’s dietary needs (M) preventing health decline due to poor sustenance (O) |  |
| **22** ^(57)^ | **22a** If healthcare professionals have knowledge and understanding of the nutritional needs of older people who are at risk of malnutrition (C) older people receive correct information, do not receive conflicting messages (M) are not advised to follow restricting diets that are potentially harmful (O) | **22e** If older people who are at greater risk of malnutrition (C), hold a combination of commonly held perceptions such as being thin is healthy and ‘snacking’ is unhealthy (M – reasoning), lack of reliable information about what a healthy diet should consist of in later life and low appetite (M- resource) can lead to poor nutritional intake and weight loss (O). |
|  | **22b** If health professionals caring for older people who are at risk of malnutrition (C), have good knowledge and understanding [through education] of how to deliver dietary advice adapted to older people’s needs (M-resource) this can ensure older people receive correct information, do not receive conflicting messages and are not advised to follow restricting diets that are potentially harmful (O). |  |
| **33** ^(68)^ | **33c** If care home staff receive appropriate training on nutrition, including special dietary needs (C), to overcome the dilemma of wanting to provide a ‘healthy diet’ whilst also responding to individual autonomy, needs and choice (M) then this can contribute towards the provision of good nutritional care (O). |  |
| **37** |  | **37b** When a first-time mother’s position on the weaning guidelines and existing weaning knowledge are acknowledged (C) greater confidence and trust in the advice given by the HV (M) can mean that weaning information is more likely to be well received and adhered to (O). |

**Practising new skills – consolidated CMOCs 13-17**

| **Consolidated CMOC 13**  **If** people had opportunities to socialise with others, practise and experience meal planning, preparation, cooking, storing and tasting new foods/dishes **then** this can result in waste less food, save time and money preparing and cooking food, increased cooking from raw ingredients and reduced intake of HFSS convenience/fast foods, healthier food choices and better-quality diet **because** of increased knowledge, skills, confidence, enjoyment and self-efficacy in ability to cook home prepared meals.  **Confidence and self-efficacy in cooking skills resulting in reduced reliance on processed and convenience HFSS foods – consolidated by outcome**  **CMOCs contributing are** **7a, 11e, 18a, 18c, 31a, 31b, 36f**  **7a** If adults were able to access practical sessions (C) that taught the importance of planning ahead, using left over ingredients and batch cooking (and storing in fridge or freezer) (C or M) increase self-efficacy in cooking skills (M) resulting in a reduction in the amount of convenience foods being eaten at home (O), to CFS more frequently, minimised time and energy (and avoid waste) (O) healthier food choices and better diet quality (O).  **11e** If parent on low income (C) are confident and enjoy cooking, are less stressed when involving young children, have a repertoire of well-known dishes with less risk of rejection when serving them (M) then this can help to develop new tastes and acceptability of these foods (O) and promote consumption of healthier, unprocessed foods (O)  **18a** If people living in areas of social deprivation have opportunities to socialise with others and learn new skills (C) confidence to cook from raw ingredients can increase (M) resulting in snacking less often due to eating more regular meals, reusing left over food, minimising food waste and reduced consumption of HFSS take away/fast **foods** (O).  **18c** **If people have opportunities to practise/ experience meal planning, preparation and cooking (C) then increased knowledge and confidence of how to cook in bulk, storing and freezing food properly, using left over meals to make new meals and preparing food from raw ingredients (M) can result in waste less food, save time and money preparing and cooking food, increased cooking from raw ingredients (O) and reduced intake of HFSS convenience/fast foods (O).**  **31a** If school-based, practical cooking skills activities for families of lower SES involve mothers [parents] as the ‘gatekeeper’ of foods purchased for/provided within the home, in preparing tasty meals (C) then this can increase skills and confidence to cook home prepared meals (M) and improve healthy eating at the family level (O)  **31b** If families of lower SES have opportunities to prepare, cook and taste new foods/ dishes (C) then this can eliminate fear of wasting food/money and improve confidence (M) to cook homemade meals from scratch and use healthier cooking practices (O)  **36f** If people most likely to rely on takeaway or fast foods rather than home-cooked meals [e.g. working overtime, those of lower educational attainment and household income, younger individuals, and men] are offered community-based cooking interventions (C) to increase practical skills and capability (self-efficacy) to cook (M) then this can reduce reliance on HFSS foods and increasing preparation and consumptions of healthier home-cooked meals (O).  **Consolidated CMOC 14**    **If** practical cooking activities for families include parents (e.g., mothers as the ‘gatekeeper’ of food provision in the home), teach the importance of planning ahead, using left over ingredients and batch cooking and focus on developing children and young people’s food and cooking skills **then** participants are more likely to feel confident to experiment, prepare a wider repertoire of dishes from scratch, avoid food waste, improve healthy eating at the family level and supports better diet quality outcomes for children that track through to adult life **because** of greater self-efficacy, knowledge, skills and confidence to cook home prepared meals, ability to shop more thriftily and easily adjust recipes to meet family preferences and possibly increased food and health motivation and cooking identity of children and young people.  **Greater self-efficacy for specific vulnerable groups – consolidated by mechanism for specific population groups (context)**  **CMOCs contributing are 7a, 7c, 7e, 31a, 42a**  **7a** If adults were able to access practical sessions (C) that taught the importance of planning ahead, using left over ingredients and batch cooking (and storing in fridge or freezer) (C or M) increase self-efficacy in cooking skills (M) resulting in a reduction in the amount of convenience foods being eaten at home (O), to CFS more frequently, minimised time and energy (and avoid waste) (O) healthier food choices and better diet quality (O).  **7c** When adults/ parents responsible for meal provisioning in the home (C) are able to; shop more thriftily (e.g. bulk buying, taking advantage of supermarket special offers), batch cook (refrigerating or freezing portion (s) for another meal), and easily adjust recipes to meet family preferences (M) then this can contribute to greater self-efficacy in one’s ability (M) and facilitate CFS (O).  **7e** If people on a limited income (C) have greater self-efficacy in their ability to cook (M) then they are more likely to feel confident to experiment, have a wider repertoire of dishes and able to fully benefit from special offers on food(O).  **31a** **If school-based, practical cooking skills activities for families of lower SES involve mothers [parents] as the ‘gatekeeper’ of foods purchased for/provided within the home, in preparing tasty meals (C) then this can increase skills and confidence to cook home prepared meals (M) and improve healthy eating at the family level (O)**  **42a** If community food initiatives that focus on developing C&YPs food and cooking skills (C) include strategies to develop knowledge and self-efficacy such as food and health motivation and cooking identity (M) then this supports better diet quality outcomes that track through to adult life (O).  **Consolidated CMOC 15**  **If** people are provided with information alongside the opportunity to practice cooking and participate in fun activities **then** this reduces reliance on HFSS convenience foods and promote increased consumption of healthier, unprocessed foods **because** of enjoyment and pleasure from cooking, reduced stress when involving young children, reduced risk that family members reject food and increased intention to cook from basic ingredients.  **Enjoyment and pleasure from cooking – consolidated by mechanism and considering different population groups (context)**  **CMOCs contributing are 11e, 14a, 30a**  **11e** If parent on low income (C) are confident and enjoy cooking, are less stressed when involving young children, have a repertoire of well-known dishes with less risk of rejection when serving them (M) then this can help to develop new tastes and acceptability of these foods (O) and promote consumption of healthier, unprocessed foods (O)  **14a** If ex heroin users are supported with learning how to shop for food and prepare meals as part of broader life skills training (C) and a return of pleasure in eating, food preparation and consumption; becoming more sociable, creative and satisfying (M) then this can result in better dietary habits and nutritional intake (O).  **30a If people are provided with information, alongside the opportunity to practice cooking and participate in fun activities (C) then this can increase the enjoyment of cooking and increase intention to cook from basic ingredients (M) and reduce reliance upon high fat, sugar, salt convenience foods (O).**  **Consolidated CMOC 16**  **If** people have an opportunity to experiment and cook new dishes and have a repertoire of well-known dishes **then** this increases the likelihood of cooking from scratch at home and consuming healthier, unprocessed foods **because** it helps to eliminate fear of wasting food and money instilled by previous personal disasters in the kitchen, improves confidence and reduces the risk of rejection when new dishes are served  **Conversely,**  **If** families on a low income do not have a safe space for experimenting and learning [eroded due to financial constraints] with food preparation and cooking **then** they are less likely to repeatedly practice developing new skills to prepare minimally processed foods **because** they fear failure or the risk of rejection when serving food, leading to wasting food and money  **Fear of failure and waste**  **CMOCs contributing to this are** **7d, 11d, 11e, 31b**  **7d** **When adults have an opportunity to experiment and cook new dishes (C) being able to fail without wasting food and money helped to overcome fear instilled by previous personal disasters in the kitchen (M)increasing the likelihood of cooking from scratch at home (O)**  **11d** If families on a low income (C) fear failure when experimenting with food preparation and cooking [SED ‘erodes the safe space for experimentation in the development of new competencies’] (M) then they are less likely to repeatedly practice developing new skills to prepare minimally processed foods (O).  **11e** If parent on low income (C) are confident and enjoy cooking, are less stressed when involving young children, have a repertoire of well-known dishes with less risk of rejection when serving them (M) then this can helping to develop new tastes and acceptability of these foods (O) and promote consumption of healthier, unprocessed foods (O)  **31b** If families of lower SES have opportunities to prepare, cook and taste new foods/ dishes (C) then this can eliminate fear of wasting food/money and improve confidence (M) to cook homemade meals from scratch and use healthier cooking practices (O)  **Consolidated CMOC 17**  **If** people from vulnerable groups lack knowledge of what constitutes healthy eating and lack time, skills and equipment to prepare and cook food at home t**hen** this can lead to a greater frequency of consumption of HFSS processed foods increasing the risk of diet-related ill health **because** of confusion about how/why to make healthy choices, competing priorities e.g., belief that immediate priority is to avoid hunger and provide foods that are filling and acceptable rather than healthy, perception that take away foods are more cost-effective and attractive alternative to cooking at home and lack of confidence to cook.  **Increased consumption of HFSS processed foods - consolidated by outcome**  **Contributing CMOCs are 5c, 6d, 10c, 11c, 17a, 36b this links with complex factors and understanding need**  **5c** **When parents lacked knowledge of what constitutes healthy eating, despite high desire to provide a healthy diet for their family (C) their confusion about how/why to make healthier choices (M) resulted in inadvertently including HFSS foods in children’s diets (O)**  **6d** If people lack the ability to cook at home (C) and lack cooking skills or confidence to cook (M) then their food choices are more limited and processed or convenience foods more the norm (O) which could result in people consuming more HFSS foods (O).  **10c** As families from South Asian communities who are required to attend mosque every day have less time for evening meal preparation (C) this can increase reliance on and consumption of HFSS snacks (O) increasing the risk of developing childhood obesity (O). [mechanism unclear]  **11c** If parents in areas of deprivation who are providing food for families (C) worry that their children may go hungry and have a strong desire/ belief that the main priority is to ensure family ‘got fed’ something filling and acceptable to them (M) rather than long term health concern (M) resulting in lower F&V and higher processed HFSS food consumption (O).  **17a** If migrants to the UK have less time for food preparation (C) due to a strong desire and motivation to optimise time spent working to support themselves, and their family in their country of origin (M) then higher consumption of cheaper, low quality fast foods (O) can increase risks of developing overweight and obesity (O).  **36b** If people from lower SE groups (C) have fewer resources, kitchen facilities and/or skills for cooking meals at home and perceive take-away foods as a more cost-effective or attractive alternative to cooking at home (M) resulting in increased frequency of consumption and reliance on take away foods [often HFSS] (O). |
| --- |

***CMOCs contributing to consolidated CMOCs 13-17***

| **Paper** | **CMOCs for reduced consumption of HFSS foods** | **CMOCs for increased consumption of HFSS foods** |
| --- | --- | --- |
| **5** ^(40)^ |  | **5c** When parents lacked knowledge of what constitutes healthy eating, despite high desire to provide a healthy diet for their family (C) their confusion about how/why to make healthier choices (M) resulted in inadvertently including HFSS foods in children’s diets (O) |
| **6** ^(41)^ |  | **6d** If people lack the ability to cook at home (C) and lack cooking skills or confidence to cook (M) then their food choices are more limited and processed or convenience foods more the norm (O) which could result in people consuming more HFSS foods (O). |
| **7** ^(42)^ | **7a** If adults were able to access practical sessions (C) that taught the importance of planning ahead, using left over ingredients and batch cooking (and storing in fridge or freezer) (C or M) increase self-efficacy in cooking skills (M) resulting in a reduction in the amount of convenience foods being eaten at home (O), to CFS more frequently, minimised time and energy (and avoid waste) (O) healthier food choices and better diet quality (O). |  |
|  | **7c** When adults/ parents responsible for meal provisioning in the home (C) are able to; shop more thriftily (e.g. bulk buying, taking advantage of supermarket special offers), batch cook (refrigerating or freezing portion (s) for another meal), and easily adjust recipes to meet family preferences (M) then this can contribute to greater self-efficacy in one’s ability (M) and facilitate CFS (O).  **7d** When adults have an opportunity to experiment and cook new dishes (C) being able to fail without wasting food and money helped to overcome fear instilled by previous personal disasters in the kitchen (M)increasing the likelihood of cooking from scratch at home (O)  **7e** If people on a limited income (C) have greater self-efficacy in their ability to cook (M) then they are more likely to feel confident to experiment, have a wider repertoire of dishes and able to fully benefit from special offers on food(O). |  |
| **10** ^(45)^ |  | **10c** As families from South Asian communities who are required to attend mosque every day have less time for evening meal preparation (C) this can increase reliance on and consumption of HFSS snacks (O) increasing the risk of developing childhood obesity (O).  [mechanism unclear] |
| **11** ^(46)^ |  | **11c** If parents in areas of deprivation who are providing food for families (C) worry that their children may go hungry and have a strong desire/ belief that the main priority is to ensure family ‘got fed’ something filling and acceptable to them (M) rather than long term health concern (M) resulting in lower F&V and higher processed HFSS food consumption (O). |
|  | **11e** If parent on low income (C) are confident and enjoy cooking, are less stressed when involving young children, have a repertoire of well-known dishes with less risk of rejection when serving them (M) then this can helping to develop new tastes and acceptability of these foods (O) and promote consumption of healthier, unprocessed foods (O) | **11d** If families on a low income (C) fear failure when experimenting with food preparation and cooking [SED ‘erodes the safe space for experimentation in the development of new competencies’] (M) then they are less likely to repeatedly practice developing new skills to prepare minimally processed foods (O). |
| **14** ^(49)^ | **14a** If ex heroin users are supported with learning how to shop for food and prepare meals as part of broader life skills training (C) and a return of pleasure in eating, food preparation and consumption; becoming more sociable, creative and satisfying (M) then this can result in better dietary habits and nutritional intake (O). |  |
| **17** ^(52)^ |  | **17a** If migrants to the UK have less time for food preparation (C) due to a strong desire and motivation to optimise time spent working to support themselves, and their family in their country of origin (M) then higher consumption of cheaper, low quality fast foods (O) can increase risks of developing overweight and obesity (O). |
| **18** ^(53)^ | **18a** If people living in areas of social deprivation have opportunities to socialise with others and learn new skills (C) confidence to cook from raw ingredients can increase (M) resulting in snacking less often due to eating more regular meals, reusing left over food, minimising food waste and reduced consumption of HFSS take away/fast foods (O). |  |
|  | **18c** If people have opportunities to practise/ experience meal planning, preparation and cooking (C) then increased knowledge and confidence of how to cook in bulk, storing and freezing food properly, using left over meals to make new meals and preparing food from raw ingredients (M) can result in waste less food, save time and money preparing and cooking food, increased cooking from raw ingredients (O) and reduced intake of HFSS convenience/fast foods (O). |  |
| **30** ^(65)^ | **30a** If people are provided with information (a recipe), alongside the opportunity to practice cooking and participate in fun activities (C) then this can increase the enjoyment of cooking and increase intention to cook from basic ingredients (M) and reduce reliance upon high fat, sugar, salt convenience foods (O). |  |
| **31** ^(66)^ | **31a** If school-based, practical cooking skills activities for families of lower SES involve mothers [parents] as the ‘gatekeeper’ of foods purchased for/provided within the home, in preparing tasty meals (C) then this can increase skills and confidence to cook home prepared meals (M) and improve healthy eating at the family level (O) |  |
|  | **31b** If families of lower SES have opportunities to prepare, cook and taste new foods/ dishes (C) then this can eliminate fear of wasting food/money and improve confidence (M) to cook homemade meals from scratch and use healthier cooking practices (O) |  |
| **36** ^(71)^ | **36f** If people most likely to rely on takeaway or fast foods rather than home-cooked meals [e.g. working overtime, those of lower educational attainment and household income, younger individuals, and men] are offered community-based cooking interventions (C) to increase practical skills and capability to cook (M) then this can reduce reliance on HFSS foods and increasing preparation and consumptions of healthier home-cooked meals (O). | **36b** If people from lower SE groups (C) have fewer resources, kitchen facilities and/or skills for cooking meals at home and perceive take away foods as a more cost-effective or attractive alternative to cooking at home (M) resulting in increased frequency of consumption and reliance on take away foods [often HFSS] (O). |
| **42** ^(77)^ | **42a** If community food initiatives that focus on developing C&YPs food and cooking skills (C) include strategies to develop knowledge and self-efficacy such as food and health motivation and cooking identity (M) then this supports better diet quality outcomes that track through to adult life (O). |  |
