# Supplementary material for: A Realist Scoping Review of Community Nutrition Interventions in the UK: Implications for the ‘Nutrition Skills for Life’ Programme
Source: J Hum Nutr Diet. 2025 Jan 8;38(1):e70008. doi: 10.1111/jhn.70008 (PMC11707723; doi:10.1111/jhn.70008)
Supplement: Supplementary file 6 — Overview of the Nutrition Skills for Life Initial Programme Theory. [file JHN-38-0-s005.docx]

**Supplementary file 6. Summary of NSFL IPT (CMOCs for HCPs/ practitioners/ group facilitators are in grey)**

|  | **Context** | **Actor** | **Mechanism** | **Outcome** |
| --- | --- | --- | --- | --- |
| Understanding community needs | Understand complex factors impacting nutrition | Organisations, practitioners | - Tailored support | - CNIs meet need - Improved nutrition |
|  |  | Community members | - Feel acknowledged |  |
|  | Community/ group discussion and interaction | Practitioners/ CNI facilitators | - Build partnership - Trusted relationships | - Engagement |
|  |  | Community members | - Trust in others - Confidence to share own experience - Acquire new skills | - Self -value, cooking self-efficacy, resilience, confidence |
|  | Education and training in motivational interviewing, how to have healthy conversations, goal setting, wider determinants of health | Healthcare professionals (HCPs) | - Critical reflection on emotions, attitudes, beliefs - Acquire knowledge, behaviour change skills and confidence | - Brief interventions delivered to meet health needs - Facilitate diet behaviour change |
| Consistent nutrition messages | Incomplete knowledge of nutrition  Poor access to reliable nutrition information  Desire (motivated) to feed family well | Community members, vulnerable groups | - Confusion - Mistaken beliefs and perceptions - Information seeking | - Low adherence to dietary guidelines - Poor nutritional intake |
|  | Knowledge and understanding of nutritional needs  Understand existing nutrition knowledge, beliefs and perceptions of others | Healthcare professionals | - Adapt dietary advice to individual needs (tailor) | - Reliable, trusted information available - address knowledge-attitude gap - safe practice |
|  |  | Vulnerable groups e.g. new parents, older adults and relatives | - Feel supported - Acquire reliable information (knowledge acquisition) - Confusion avoided | - Improved nutrition |
|  | Retail outlets and public sector settings provide unhealthy foods that are more affordable | Organisations, retail sector, public sector, CNI facilitators | - Misalignment of message - Perception unhealthy foods are ‘treats’ - Perception healthy foods are unaffordable | - Conflicting messages - Efforts to encourage healthy foods undermined |
|  |  | Community members | - Confusion - Perception unhealthy foods are ‘treats’ - Perception healthy foods are unaffordable |  |
|  | Nutrition education & practical cooking skills development opportunities  Include digital technology and social media | New parents, young adults, people with long term conditions | - Motivation - Learning nutrition, health and skills | - Dietary behaviour change - Change daily routines - Reduce food waste - Reduce food spending |
| Knowledgeable, skilled, confident practitioners | HCPs receive training and education in nutrition, behaviour change techniques, goal setting | Healthcare professionals | - Confidence - Self-efficacy to raise sensitive topics - Empowered to provide support - Alleviate fear | - Dietary advice and support provided for vulnerable groups - More healthy conversations - Relationships with community members are protected |
|  | Families supported by trained HCPs to set goals for dietary change that they are able to achieve | People during pregnancy and early parenthood | - Trusted advice - Confidence - Self-efficacy to make incremental dietary changes - Sense of ownership - Accomplishment and pride when goals are met. | - Dietary behaviour change supported |
|  | Staff have nutrition knowledge, understand nutritional needs of older adults, alert for signs of malnutrition (undernutrition) | Staff, carers | - Adapt dietary advice to meet need (tailor) | - Optimum nutritional care |
|  |  | Older people, relatives | - Confusion avoided - Perceptions addressed - Relatives proactively contribute to care |  |
| Practising new skills | Opportunities to socialise with others and practise food preparation skills | Vulnerable groups | - Learn (acquire knowledge) to shop, prepare, cook, store food - Confidence to cook - Enjoyment and pleasure - Self-efficacy to prepare and cook meals | - Reduce food waste, time and money - Increase cooking from raw/ basic ingredients - Reduced high fat, sugar, salt (HFSS) foods - Healthier food choices & better-quality diet |
|  | Involve parents as ‘gatekeepers’ of food provision  Teach planning ahead, using left over ingredients, batch cooking  Develop children and young people’s food and cooking skills | Parents, children and young people (CYP) | - Self-efficacy, knowledge, skills and confidence to cook - Shop thriftily - Adjust recipes - Motivation and cooking identity (CYP) | - Increased confidence to experiment - Avoid food waste - Healthy eating at family level - Better diet quality |
|  | Information, opportunities to practise cooking and fun activities | Vulnerable groups | - Enjoyment and pleasure - Reduced stress - Reduced risk of rejection | - Reduced reliance on HFSS foods - Increased consumption of healthier unprocessed foods |
|  | Opportunity to experiment, cook new dishes, develop repertoire of well-known dishes | Vulnerable groups | - Eliminates fear of failure and waste - Confidence to cook - Reduced risk of rejection | - Increased likelihood of practising cooking at home - Increased consumption of healthier unprocessed foods |
|  | Lack knowledge of healthy eating, time, skills and equipment to prepare food at home | Vulnerable groups | - Confusion about how/why healthy choices - Competing priorities - Beliefs and perceptions | - Increased consumption of HFSS foods - Increased risk of diet related ill health |
