# Supplementary material for: A Realist Scoping Review of Community Nutrition Interventions in the UK: Implications for the ‘Nutrition Skills for Life’ Programme
Source: J Hum Nutr Diet. 2025 Jan 8;38(1):e70008. doi: 10.1111/jhn.70008 (PMC11707723; doi:10.1111/jhn.70008)
Supplement: Supplementary file 7 — Relevant substantive theory. [file JHN-38-0-s006.docx]

| **Social Change Theory** | **Diffusion of Innovations Theory** | **Social Cognitive Theory** |
| --- | --- | --- |
| External environment influences community goals, norms, values and organisations which influence social norms (shared rules and expectations) regarding health behaviours, which brings about behaviour change at the individual level.   - Social **norms** that lead to unhealthy behaviours can be replaced with social norms that support healthier behaviours - Health improvements are best achieved by altering community rather than individual norms - Communities comprise political, economic, health, education, communication, religious, recreational and social welfare subsystems; voluntary groups and social movement groups. - **Community system** is stable - Stimuli from **external environment** can influence norms and values within system - **Vested interests** (e.g. food industry) aim to preserve the status quo - **Social movements** (e.g., those campaigning for food system change) arise to counter vested interests. - **Community developments** can occur with the aim of changing community behaviours - External forces (e.g., government policies) use **locality development** or social planning theories to bring about change - Subsystem level- **community organisations** work together to achieve change - Additional policies (e.g., healthy meals regulations in schools) are imposed - **Leadership roles**- organisations spread change to other groups through **organisational development** and **diffusion** via social networks - People are exposed to changing norms which are reinforced by influential **role models** - **Collective action** and changes in the **social environment** bring about new norms - Widespread individual **behaviour change** is likely to occur   *Adapted from Michie et al p356* | Explains the process by which people or social groups adopt or reject a new idea, behaviour or object (e.g., dietary advice, healthy eating policy, healthy lifestyle app).  Specifies numerous mechanisms through which adoption or rejection is achieved, and factors that facilitate adoption.   - ‘Diffusion’ is the process by which **innovations** are communicated in different ways over **time**, to people within a **social system** - **An innovation is defined as an idea, behaviour or object that is seen as new by a person or group that may adopt it.** - Technological innovation, the original focus of the theory was conceptualised as having **hardware** **aspec**t and **software aspect** components. Software components are the informational basis of the tool. - Technology is defined as the mechanism that explains the cause and effect relationship involved in reducing the **uncertainty** of achieving a goal - Certain characteristics influence rate of adoption of an innovation; the greater the perceived **relative advantage** of the new innovation over the idea that it supersedes; the greater the perceived **compatibility** with the needs and values of the adopters; the lower the perceived **complexity** (perceived difficulty of understanding and usage) of the innovation; the greater the **trialability** (extent it can be experimented with); the greater the **observability** (outcomes are visible) and the greater potential for **re-invention** (ability to evolve to fit people’s needs), the faster the adoption rate/ - Communication channels, to pass on information about the innovation includes **mass media channels** – to transmit quickly to a wide audience and **interpersonal channels** – face-to- face communication is a more effective way**,** especially if from **near-peers** through new adopters **modelling** the behaviour of previous adopters. - If communicators and potential adopters are **homophilous** (share the same attributes) within the same social system, communication is more effective - Adoption of the new innovation occurs in a 5-stage process **knowledge, persuasion, decision, implementation** and **confirmation** - Characteristics of adopters determine the time required for diffusion, 5 categories described are **innovators, early adopters, early majority, late majority, laggards**   *Adapted from Michie et al p105* | Provides a framework for understanding human thought and behaviour. Proposes that behaviour, the environment and personal and cognitive factors all interact as determinants of each other, referred to as **reciprocal determinism.**   - People are not simply shaped by their environment, they are active participants in it. While the environment in which people are born, live, learn, work and age contribute to behaviour, the individual person (and the way they process/their cognition) is just as important. - Social learning and knowledge acquisition occur as a result of people observing others - Human functioning is described in terms of 5 basic capabilities:   **symbolising capability-** transforming experience into mental models that can guide future behaviour and provide meaning to experience**;**  **forethought capability-** regulate behaviour on the basis of the future e.g. goal setting, action planning and motivation & guidance of actions based on anticipated outcomes**;**  **vicarious capability** -ability to learn through observation/ imitation/ modelling others’ behaviours and attitude;  **self-regulatory capability-** regulate own intentions and behaviours, adjusting behaviour and/or environment to meet personal standards,  **self-reflective capability –** analyse own experiences, thoughts and knowledge and verify /modify them to generate new ideas, adjust thoughts and act accordingly   - **Self-efficacy – people’s judgements on their ability to cope effectively in different circumstances, or master a particular skill (i.e., perceived self-efficacy) a type of self-reflective thought, is the most influential upon behaviour.** - **Perceptions of self-efficacy influence people’s choice of action, the effort and perseverance they invest in action and the anxiety or confidence with which they approach actions.** - Actions that are influenced by perceptions of self-efficacy result in either success or failures, which will be reflected upon to inform future judgements of self-efficacy   *Adapted from Michie et al p359* |
